# Ascorbic Acid-Initiated Tandem Radical Cyclization of *N*-Arylacrylamides to Give 3,3-Disubstituted Oxindoles

**DOI:** 10.3390/molecules200915631

**Published:** 2015-08-27

**Authors:** Sheng Liu, Pi Cheng, Wei Liu, Jian-Guo Zeng

**Affiliations:** 1 Hunan Co-Innovation Center for Utilization of Botanicals Functional Ingredients, Hunan Agricultural University, Changsha 410128, China; E-Mails: shengliu21@163.com (S.L.); lwhncs618@163.com (W.L.); ginkgo@world-way.net (J.-G.Z.); 2 Pre-State Key Laboratory for Germplasm Innovation and Utilization of Crop, Hunan Agricultural University, Changsha 410128, China

**Keywords:** *N*-arylacrylamides, 3,3-disubstituted oxindoles, tandem cyclization, ascorbic acid, 4-nitroaniline

## Abstract

An ascorbic acid-promoted and metal-free tandem room temperature cyclization of *N*-arylacrylamides with 4-nitrobenzenediazonium generated *in situ* was developed. This reaction proceeds smoothly through a radical mechanism and provides an environmentally friendly alternative approach to biologically active 3-alkyl-3-benzyloxindoles, avoiding the use of excess oxidants and light irradiation.

## 1. Introduction

**Scheme 1:**
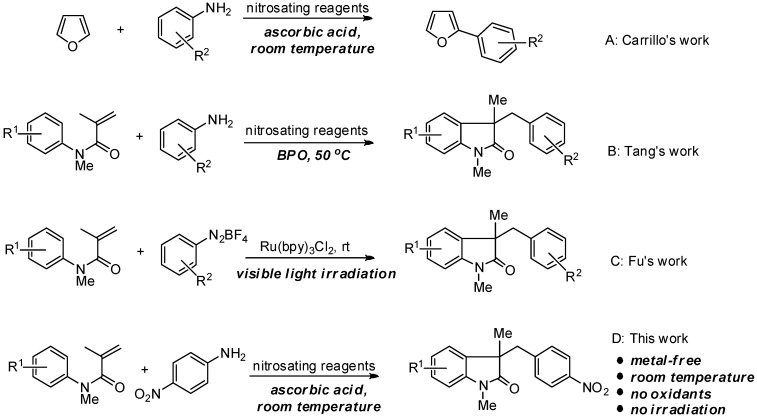
(**A**) Ascorbic acid-initiated direct C-H arylation; (**B**,**C**) Previous strategies for synthesis of 3-alky-3-benzyloxindoles; (**D**) Our strategy for synthesis of 3-alkyl-3-benzyloxindoles.

Ascorbic acid, commonly known as a food additive, has non-food uses in chemistry. For example, it is used as a reductant in photographic developer solutions [1]. In addition, it is a safe reducing agent in organic synthesis [2,3] and can promote quinone redox cycling [4,5,6,7]. Recently, Carrillo and co-workers reported that ascorbic acid could act as a radical initiator for the direct C-H arylation of arenes with anilines nitrosated *in situ* [8]. In this report, ascorbic acid was able to reduce the arenediazonium ion generated *in situ* by a single-electron transfer (SET) process to afford aryl radicals as a coupling partner with arenes such as furan (Scheme 1A). This Meerwein/Sandmeyer-type reaction promoted by ascorbic acid was mild and environmentally friendly, not requiring heat or irradiation.

*N*-arylacrylamides are a type of an ideal radical acceptor which has been proved to be an platform for construction of 3,3-disubstituted oxindoles [9], important biologically active heterocycles, via a tandem radical cyclization process. The electron-deficient carbon-carbon double bond of *N*-arylacrylamides could accept the addition from different radical species such as *C*-radicals (including alkyl [10,11], acyl [12] and aryl radicals [13,14]), *N*-radicals [15], *Si*-radicals [16] and *P*-radicals [17]. Although the tandem reactions between *N*-arylacrylamides and radical species are well established, the reaction conditions for generation of the radical species were generally harsh or violent. In some cases, transition-metal and strong oxidants such as *t*-butyl hydroperoxide (TBHP) [18] or di-*t*-butyl peroxide (DTBP) [19] were required. As to the generation of aryl radicals, only two examples were reported. Tang and Wang [13] reported a straightforward carboarylation reaction of *N*-arylacrylamides and aniline via a tandem Meerwein arylation/C-H cyclization to construct the 3-benzyl-3-alkyloxindole scaffold in the presence of benzoyl peroxide (BPO) and *tert*-butyl nitrite (*t*-BuONO) (Scheme 1B). Although the reaction temperature was 50 °C, an additional 5 mol % of oxidant BPO was still required as initiator. Almost at the same time, Fu, Zou, and co-workers [14] developed a visible light-induced photocatalytic tandem aryl radical cyclization between commercially available arenediazonium salts and *N*-arylacrylamides to afford 3-benzyl-3-alkyloxindoles at room temperature (Scheme 1C). The reaction is mild but visible light and the photocatalyst Ru(bpy)_3_Cl_2_ were needed to initiate the reaction. Inspired by Carrillo’s pioneering works, we herein report a procedure for the synthesis of 3,3-disubstituted oxindoles via ascorbic acid-initiated direct carboarylation of *N*-arylacrylamides with aniline nitrsated *in situ*. (Scheme 1D). This newly developed method avoids the use of excess oxidants and light irradiation.

## 2. Results and Discussion

In our reaction condition optimization process, *N*-methyl-*N*-phenylacrylamide (**1a**) and 4-nitroaniline (**2a**) were selected as model substrates. As shown in Table 1, when *N*-methyl-*N-*phenylacrylamide and 1.5 equiv of 4-nitroaniline in acetonitrile (MeCN) were treated with ascorbic acid (0.1 equiv) in the presence of 1.5 equiv of various nitrosating reagents (Table 1, entry 1–3), the best nitrosating reagents was found to be *t*-BuONO (Table 1, entry 1) and the desired cyclization product 3-methyl-3-(4′-nitrobenzyl) oxindole (**3a**) was obtained in 21% isolated yield. Further solvent screening showed that dimethyl sulfoxide (DMSO) was a suitable solvent for the cyclization reaction (Table 1, entry 4) and other solvents such as DMF and DCE led to decreased yields of compound **3a** (Table 1, entries 5–6). 

**Table 1:** Reaction Conditions Screening ^a^. 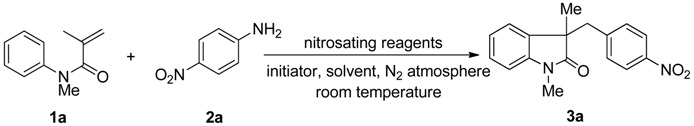

Entry	Initiator 10 mol %	Nitrosating Reagent	1a/2a/Nitrosating Reagent	Solvent	Yields% ^b^
1	-	*t*-BuONO	1:1.5:1.5	MeCN	21
2	-	*i*-AmONO	1:1.5:1.5	MeCN	13
3	-	NaNO_2_	1:1.5:1.5	DMSO	9
4	-	*t*-BuONO	1:1.5:1.5	DMSO	19
5	-	*t*-BuONO	1:1.5:1.5	DMF	10
6	-	*t*-BuONO	1:1.5:1.5	DCE	12
7	-	*t*-BuONO	1:3:3	MeCN	33
8	Ascorbic acid	*t*-BuONO	1:3:3	MeCN/DMSO ^c^	63
9	-	*t*-BuONO	1:3:3	MeCN/DMSO ^c^	36
10 ^d^	Ascorbic acid	*t*-BuONO	1:3:3	MeCN/DMSO ^c^	60
11 ^e^	Ascorbic acid	*t*-BuONO	1:3:3	MeCN/DMSO ^c^	42

^a^ Reaction conditions: **1a** (0.25 mmol), 4-nitroaniline (1.5 or 3.0 equiv), initiator (10 mol %) and nitrosating reagents in solvent (5 mL) was stirred for 24 h at room temperature under nitrogen atmosphere; ^b^ Isolated yields; ^c^ V_MeCN_:V_DMSO_ = 4:1; ^d^ Reaction was carried out in the dark; ^e^ Reaction was carried out in open flask. *t*-BuONO = *tert*-butyl nitrite, *i*-AmONO = *iso-*armyl nitrite.

To enhance the yield of **3a**, an excess of 4-nitroaniline and *t*-BuONO was found to be required. Target compound **3a** was obtained in 33% isolated yield when the reaction was carried out in 3.0 equiv of 4-nitroaniline and *t*-BuONO (Table 1, entry 7). Although the tandem cyclization reaction proceeded smoothly to give desired compound, the yield was poor. Based on Carrillo’s findings, ascorbic acid was an ideal radical initiator to promote the homolytic rupture of diazo intermediate generated *in situ*. In terms of our cyclization reaction, 10 mol % of ascorbic acid was found to be able to promote the outcome of compound **3a** in a mixture solvents of MeCN and DMSO (4:1 volume ratio) (Table 1, entry 8) due to the poor solubility of ascorbic acid in MeCN. As a control, the reaction was carried out in an MeCN/DMSO mixture solvent without ascorbic acid, which resulted in a decreased yield of compound **3a** (36% yield, Table 1, entry 9). To study the effect of visible light from the experimental environment, another control reaction was conducted in the dark. This proceeded well to afford the compound **3a** in 60% yield (Table 1, entry 10), which indicated that visible light was not necessary for the cyclization reaction. In addition, the reaction was tested under open air conditions, which led to a decrease in the yield (Table 1, entry 11), because oxygen can react with the radicals formed during the reaction.

**Scheme 2:**
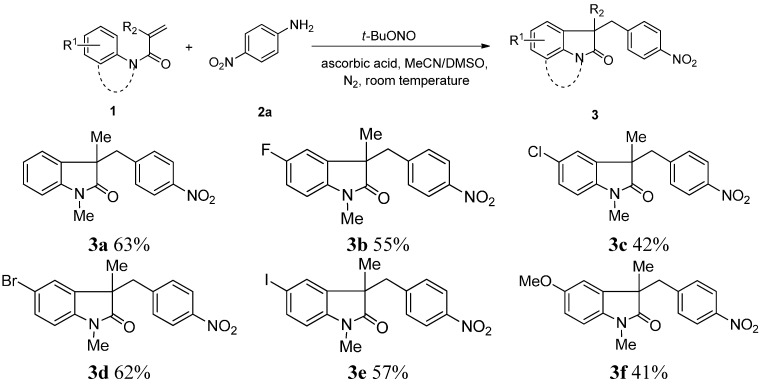

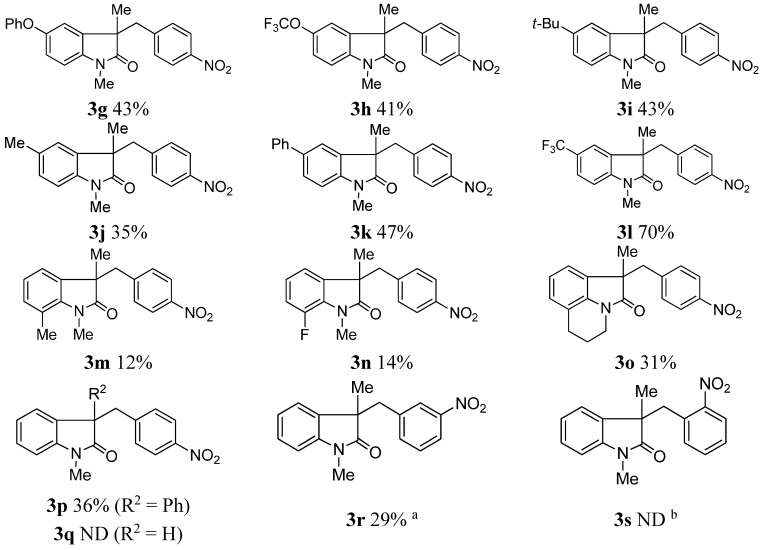
The scope of the proposed *N*-arylacrylamine synthesis. Yields are for the isolated product after silica gel column chromatography.

With optimized conditions in hand, we turned our study to screening the the substrate scope. Firstly, we tested the effect of the substituent group on the phenyl ring of substrate **1**. As shown in Scheme 2, the substituents on the phenyl ring possessed significant effect on the yields of cyclization products. Halogen atoms (F, Cl, Br, I) on the ring of compounds **1** were compatible with the cyclization reaction, and the corresponding products **3b**–**3e** were obtained in moderate yields. Interestingly, the Br and I group remained intact in the C-H functionalization process, which thereby facilitated additional modifications at the halogenated positions. Next, a set of *N*-arylacrylamides with electron-donating groups (R^1^ = 4-MeO-, 4-PhO-, 4-CF_3_O-, 4-*t*-Bu- and 4-CH_3_-) on the phenyl ring were evaluated as reaction substrates, which resulted in decreased yields of the corresponding cyclized compounds **3f**–**3k**. In contrast, replacement of these electron-donating groups with an electron-withdrawing group such as CF_3_ significantly promoted the formation of the desired compound **3l**. In addition, a significantly decreased yield of the desired compounds **3m**–**3o** was observed when *ortho*-substituted *N*-arylacrylamides were used as reaction substrates. We also tested the effect of group R^2^ (R^2^ = Ph or H) of compounds **1** on the tandem cyclization reaction and found that **3p** (R^2^ = Ph) was obtained in 36% yield, while compound **3q** (R^2^ = H) was not detected under the present reaction conditions. Finally, 3-nitroaniline and 2-nitroaniline were used instead of 4-nitroniline, which led to a modest 29% yield of compound **3r** and a complex reaction mixture, respectively.

*Reagents and conditions*: **1** (0.5 mmol), **2a** (1.5 mmol), *t*-BuONO (1.5 mmol), ascorbic acid (10 mol %), and MeCN/DMSO (10 mL) in nitrogen atmosphere at room temperature for 24 h. ^a^ 3-nitroaniline was used instead of 4-nitroaniline. ^b^ 2-nitroaniline was used instead of 4-nitroaniline. ND: not detected.

After the *N*-arylacrylamide scope screening, we attempted to explore the scope of substituents on the aniline moiety under the optimized reaction conditions. Unfortunately, replacement of 4-nitroaniline with aniline, 4-chloroaniline or 4-trifluoromethylaniline all resulted in a very complex reaction mixture from which the *N*-arylacrylamides were recovered, and only trace of the desired cyclization compounds could be detected through LC-MS analysis.

**Scheme 3:**
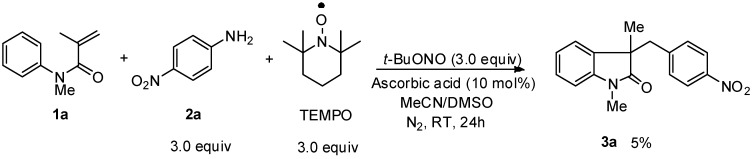
Control experiment.

According to Tang’s previous work [13], the cyclization reaction might proceed through a radical pathway. To gain a further insight the possible mechanism in present research, we added 2,2,6,6-tetramethylpiperidine-1-oxy radical (TEMPO) to the reaction mixture (Scheme 3) to trap the 4-nitrophenyl radical, and the resulting cyclization compound **3a** was isolated in only 5% yield.

**Scheme 4:**
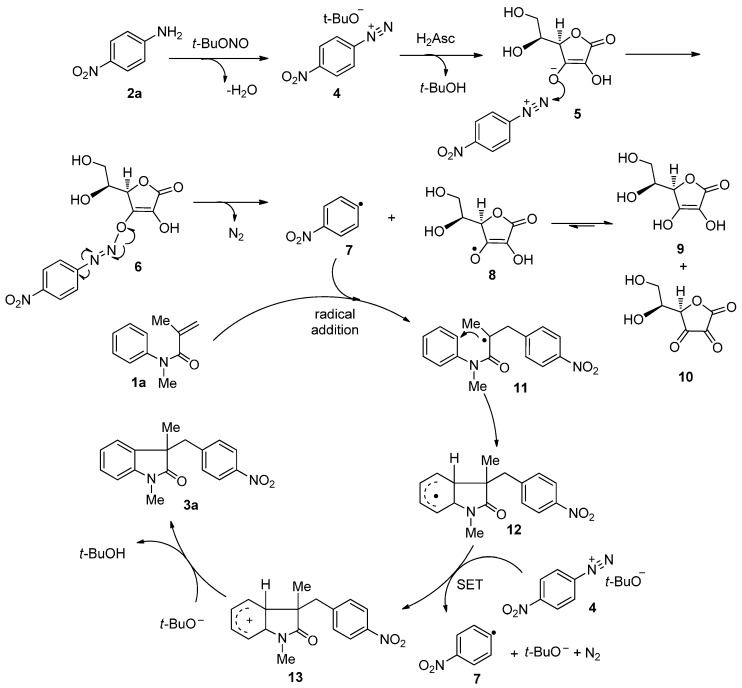
Possible cyclization mechanism.

On the basis of previous reports and the above experimental results, a tentative mechanism is proposed in Scheme 4. First, *tert*-butyl nitrite reacts with 4-nitroaniline to yield diazomium salt **4**, which is partly protonated by ascorbic acid (H_2_Asc) to afford the ascorbate diazomium salt **5**. The nucleophilic addition of ascorbate to the diazonium moiety provides diazoether **6** which was previously isolated by Brown and co-workers [20].

At this step, the strong electron-withdrawing ability and conjugative effect of the nitro group can facilitate the addition of nitrogen-nitrogen triple bonds and successive diazoether hemolytic rupture. Thus, we suggest that the nucleophilic addition and homolytic rupture steps are more difficult when 4-nitroaniline was replaced by other aniline derivatives under our reaction conditions, which finally resulted in a complex reaction mixture. Next, diazomium salt **5** is reduced by ascorbate to generate nitrogen, ascorbyl radical **8** and the aryl radical **7**. Ascorbyl radical **8** tends to dismutate into dehydroascorbic acid **10** and ascorbic acid **9**, which can reduce another arenediazonium ion [21]. On the other hand, aryl radical **7** undergoes an addition to the carbon-carbon double bond of *N*-arylacrylamide **1a** to generate the alkyl radical **11**. The intermolecular cyclization of radical **11** gives another radical intermediate **12**, which loses one electron to reduce the diazonium salt **5** molecule to generate carbocation **13** and another aryl radical **7**. The final step is deprotonation of carbocation **13** by the *tert*-butoxide anion that generated in the reaction system to yield the desired cyclization compound **3a**.

**Scheme 5:**
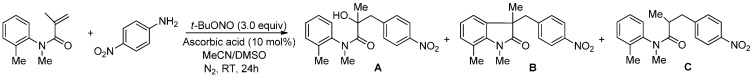
Byproducts generated with *ortho*-substituted *N*-arylacrylamide as substrate.

Based on the possible tandem cyclization mechanism, we attempted to explain why significant decreased yield of desired compounds were observed when the *ortho*-substituted *N*-arylacrylamides were used as reaction substrates (Scheme 2, **3m**–**3n**). We were interested in the byproduct generated in the reaction system. According to the HPLC-Q-TOF analysis of the crude product from the *ortho*-substituted cases, α-hydroxyl amide derivative **A** (Scheme 5) and simple C-C double bond Meerwein radical addition product **C** were detected as major byproducts (see Supplementary Materials). Due to the steric effect, the intermolecular cyclization to target compound **B** (compound **3m**) was possibly not favored. Thus, radical intermediate **11** (Scheme 4) could be oxidized by trace oxygen in the reaction system to provide byproduct **A** or be quenched to afford byproduct **C**.

## 3. Experimental Section

### 3.1. General Information

All reactions were carried out under nitrogen atmosphere unless otherwise stated. ^1^H-NMR (400 MHz) and ^13^C-NMR (100 MHz) spectra were obtained at 25 °C with CDCl_3_ as solvent and TMS as internal standard. HRMS data were obtained in the ESI mode on an Agilent 6530 Q-TOF/MS system. For flash chromatography silica gel (200–300 mesh) was employed.

### 3.2. Typical Procedure for Ascorbic Initiated Tandem Cyclization of N-Arylacrylamines *1* with 4-Nitroniline

A solution of *N*-arylacrylamine **1** (0.5 mmol), 3.0 equiv of 4-nitroaniline in MeCN (8 mL) was stirred under a nitrogen atmosphere. Then *t*-BuONO (3.0 equiv.) was added to the solution under nitrogen and stirred for 10 min. After the color of the reaction mixture turned to brown in about 20 min, a solution of ascorbic acid (0.1 equiv) in DMSO (2 mL) was added to the reaction mixture slowly under nitrogen. After the reaction was completed in 24 h, the resulting mixture was poured to water (50 mL) and extracted with dichloromethane (20 mL × 3). The combined organic solution was then washed with water (20 mL × 3) and dried over MgSO_4_. The solvent were removed *in vacuo* and the residue was purified by flash column chromatography (SiO_2_) with hexane/acetone (20:1) to give target compounds **3** as pale yellow amorphous powders.

### 3.3. Physical, Analytical and Spectral Data

*1,3-Dimethyl-3-(4-nitrobenzyl)indolin-2-one* (**3a**). ^1^H-NMR (CDCl_3_): δ 7.86 (d, *J* = 8 Hz, 2H), 7.22–7.16 (m, 2H), 7.05(t, *J* = 8 Hz, 1H), 6.97 (d, *J* = 8 Hz, 2H), 6.60 (d, *J* = 12 Hz, 1H), 3.26 (d, *J* = 16 Hz, 1H), 3.07 (d, *J* = 16 Hz, 1H), 2.95 (s, 3H), 1.49 (s, 3H). ^13^C-NMR (CDCl_3_): δ 179.2, 146.8, 144.1, 143.0, 132.1, 132.1 (overlapped), 130.6, 128.4, 123.0, 122.7, 122.7 (overlapped), 122.5, 108.2, 50.0, 44.2, 26.0, 23.3. HRMS (ESI^+^): calcd 297.1234 for C_17_H_17_N_2_O_3_ [M + H]^+^; found, 297.1232.

*5-Fluoro-1,3-dimethyl-3-(4-nitrobenzyl)indolin-2-one* (**3b**). ^1^H-NMR (CDCl_3_): δ 7.90 (d, *J* = 8.0 Hz, 2H), 7.00 (d, *J* = 8.0 Hz, 2H), 7.01–6.7 (m, 1H), 6.89 (td, *J* = 8.8, 2.4 Hz, 1H), 6.53 (dd, *J* = 8.8, 4.4 Hz, 1H), 3.29 (d, *J* = 12.8 Hz, 1H), 3.05 (d, *J* = 12.8 Hz, 1H), 2.96 (s, 3H), 1.50 (s, 3H). ^13^C-NMR (CDCl_3_): δ 178.9, 159.3 (d, ^1^*J*_F-C_ = 240 Hz), 147.0, 143.7, 139.0, 133.8 (d, ^3^*J*_F-C_ = 7.7 Hz), 130.6, 130.6 (overlapped), 123.0, 123.0 (overlapped), 114.7 (d, ^2^*J*_F-C_ = 23 Hz), 111.2 (d, ^2^*J*_F-C_ = 25 Hz), 108.8 (d, ^3^*J*_F-C_ = 8.0 Hz), 50.6 (d, ^4^*J*_F-C_ = 1.3 Hz), 44.2, 26.2, 2.3. HRMS (ESI^+^): calcd 315.1139 for C_17_H_16_FN_2_O_3_ [M + H]^+^; found, 315.1124.

*5-Chloro-1,3-dimethyl-3-(4-nitrobenzyl)indolin-2-one* (**3c**). ^1^H-NMR (CDCl_3_): δ 7.91 (d, *J* = 8.0 Hz, 2H), 7.21 (d, *J* = 2.0 Hz, 1H), 7.17 (dd, *J* = 8.4, 2.0 Hz, 1H), 7.00 (d, *J* = 8.0 Hz, 2H), 6.54 (d, *J* = 8.4 Hz, 1H), 3.29 (d, *J* = 12 Hz, 1H), 3.06 (d, *J* = 12 Hz, 1H), 2.95 (s, 3H), 1.51 (s, 3H). ^13^C-NMR (CDCl_3_): δ 178.7, 147.0, 143.6, 141.7, 133.9, 130.6, 130.6 (overlapped), 128.4, 128.1, 123.6, 123.0, 123.0 (overlapped), 109.2, 50.4, 44.2, 26.2, 23.3. HRMS (ESI^+^): calcd 331.0844 for C_17_H_16_ClN_2_O_3_ [M + H]^+^; found, 331.0853.

*5-Bromo-1,3-dimethyl-3-(4-nitrobenzyl)indolin-2-one* (**3d**). ^1^H-NMR (CDCl_3_): δ 7.91 (d, *J* = 8.8 Hz, 2H), 7.36 (d, *J* = 2.0 Hz, 1H), 7.32 (dd, *J* = 8.4, 2.0 Hz, 1H), 7.01 (d, *J* = 8.4 Hz, 2H), 6.41 (d, *J* = 8.4 Hz, 1H), 3.29 (d, *J* = 12.8Hz, 1H), 3.07 (d, *J* = 12.8 Hz, 1H), 2.96 (s, 3), 1.51 (s, 3H).^13^C-NMR (CDCl_3_): δ 178.4, 146.8, 143.5, 142., 134.2, 131.2, 130.5, 130.5 (overlapped), 126.2, 122.8, 122.8 (overlapped), 115.1, 109.6, 50.2, 44.0, 26.0, 23.1. HRMS (ESI^+^): calcd 375.0339 for C_17_H_16_BrN_2_O_3_ [M + H]^+^; found, 375.0338.

*5-Iodo-1,3-dimethyl-3-(4-nitrobenzyl)indolin-2-one* (**3e**). ^1^H-NMR (CDCl_3_): δ 7.92 (d, *J* = 8.8 Hz, 2H), 7.53 (s, 1H), 7.52–7.51 (m, 1H), 7.00 (d, *J* = 8.8 Hz, 2H), 6.41 (d, *J* = 8.8 Hz, 1H), 3.27 (d, *J* = 12.8 Hz, 1H), 3.06 (d, *J* = 12.8 Hz, 1H), 2.95 (s, 3H), 1.51 (s, 3H). ^13^C-NMR (CDCl_3_): δ 178.3, 146.9, 143.5, 142.7, 137.1, 134.6, 131.8, 130.5, 130.5 (overlapped), 122.8, 122.8 (overlapped), 122.7, 110.2, 50.0, 44.0, 26.0, 23.1. HRMS (ESI^+^): calcd 423.0200 for C_17_H_16_IN_2_O_3_ [M + H]^+^; found, 423.0209.

*5-Methoxy-1,3-dimethyl-3-(4-nitrobenzyl)indolin-2-one* (**3f**). ^1^H-NMR (CDCl_3_): δ 7.88 (d, *J* = 8.8 Hz, 2H), 7.00 (d, *J* = 8.8 Hz, 2H), 6.83 (d, *J* = 2.4 Hz, 1H), 6.70 (dd, *J* = 8.4, 2.4 Hz, 1H), 6.51 (d, *J* = 8.4 Hz, 1H), 3.79 (s, 3H), 3.26 (d, *J* = 12.8 Hz, 1H), 3.03 (d, *J* = 12.8 Hz, 1H), 2.93 (s, 3H), 1.49 (s, 3H). ^13^C-NMR (CDCl_3_): δ178.8, 156.1, 146.8, 144.2, 136.6, 133.6, 130.6, 130.6 (overlapped), 122.8, 112.1, 111.0, 108.5, 55.9, 50.5, 44.2, 26.1, 23.4. HRMS (ESI^+^): calcd 327.1339 for C_18_H_19_N_2_O_4_ [M + H]^+^; found, 327.1340.

*1,3-Dimethyl-3-(4-nitrobenzyl)-5-phenoxyindolin-2-one* (**3g**). ^1^H-NMR (CDCl_3_): δ 7.93 (d, *J* = 8.4 Hz, 2H), 7.37 (t, *J* = 8.4 Hz, 2H), 7.12 (td, *J* = 7.6, 0.8 Hz, 1H), 7.04 (d, *J* = 8.4 Hz, 2H), 6.96–6.90 (m, 4H), 6.61 (d, *J* = 8.4 Hz, 1H), 3.32 (d, *J* = 12.8 Hz, 1H), 3.04 (d, *J* = 12.8 Hz, 1H), 3.02 (s, 3H), 1.51 (s, 3H). ^13^C-NMR (CDCl_3_): δ179.0, 158.4, 152.4, 146.8, 144.0, 138.9, 133.7, 130.5, 130.5 (overlapped), 129.8, 129.8 (overlapped), 122.9, 122.8, 122.8 (overlapped), 119.6, 117.6, 1176 (overlapped), 115.8, 108.8, 50.4, 43.9, 26.1, 23.3. HRMS (ESI^+^): calcd 389.1496 for C_23_H_21_N_2_O_4_ [M + H]^+^; found, 389.1501.

*1,3-Dimethyl-3-(4-nitrobenzyl)-5-(trifluoromethoxy)indolin-2-one* (**3h**). ^1^H-NMR (CDCl_3_): δ 7.89 (dd, *J* = 8.8, 2.0 Hz, 2H), 7.12 (s, 1H), 7.07 (brd, *J* = 7.6 Hz, 1H), 7.99 (d, *J* = 8.4 Hz, 2H), 6.61 (d, *J* = 8.4 Hz, 1H), 2.99 (s, 3H), 1.53 (s, 3H). ^13^C-NMR (CDCl_3_): δ178.8, 146.8, 144.7 (d, ^2^*J*_F-C_ = 28.7 Hz), 143.4, 141.6, 133.5, 130.4, 130.4 (overlapped), 122.8, 122.8 (overlapped), 121.5, 120.6 (q, ^1^*J*_F-C_ = 255 Hz), 117.2, 108.6, 50.3, 44.0, 26.1, 22.9. HRMS (ESI^+^): calcd 381.1057 for C_18_H_16_F_3_N_2_O_4_ [M + H]^+^; found, 381.1047.

*5-(tert-Butyl)-1,3-dimethyl-3-(4-nitrobenzyl)indolin-2-one* (**3i**). ^1^H-NMR (CDCl_3_): δ 7.89 (d, *J* = 8.8, 2.0 Hz, 2H), 7.23–7.20 (m, 2H), 6.99 (dd, *J* = 8.8, 2.0 Hz, 2H), 6.55 (dd, *J* = 8.8, 1.2 Hz, 1H), 3.25 (dd, *J* = 12.8, 1.6 Hz, 1H), 3.10 (d, *J* = 12.8 Hz, 1H), 2.98 (d, *J* = 1.6 Hz, 3H), 1.52 (d, *J* = 2.0 Hz, 3H), 1.34 (brs, 9H). ^13^C-NMR (CDCl_3_): δ 179.2, 146.7, 145.7, 144.2, 140.5, 131.6, 130.6, 130.6 (overlapped), 124.6, 122.6, 122.6 (overlapped), 120.4, 107.5, 50.0, 44.2, 34.6, 31.6, 31.6, 31.6 (overlapped), 26.0, 23.0. HRMS (ESI^+^): calcd 353.1860 for C_21_H_25_N_2_O_3_ [M + H]^+^; found, 353.1877.

*1,3,5-Trimethyl-3-(4-nitrobenzyl)indolin-2-one* (**3j**). ^1^H-NMR (CDCl_3_): δ 7.88 (d, *J* = 8.8 Hz, 2H), 7.03 (s, 1H), 6.98–6.96 (1H, m, overlapped), 6.97 (d, *J* = 8.8 Hz, 2H), 6.50 (d, *J* = 8.0 Hz, 1H), 3.25 (d, *J* = 12.8 Hz, 1H), 3.06 (d, *J* = 12.8 Hz, 1H), 2.94 (s, 3H), 2.36 (s, 3H), 1.49 (s, 3H). ^13^C-NMR (CDCl_3_): δ 179.1, 146.8, 144.3, 140.7, 132.2, 132.1, 130.6, 130.6 (overlapped), 128.7, 123.9, 122.8, 122.8 (overlapped), 108.0, 50.1, 44.3, 26.1, 23.4, 21.3. HRMS (ESI^+^): calcd 311.1390 for C_18_H_19_N_2_O_3_ [M + H]^+^; found, 311.1383.

*1,3-Dimethyl-3-(4-nitrobenzyl)-5-phenylindolin-2-one* (**3k**). ^1^H-NMR (CDCl_3_): δ 7.91 (d, *J* = 8.8, Hz, 2H), 7.59 (d, *J* = 7.6 Hz, 2H), 7.50–7.45 (m, 4H). 7.38 (t, *J* = 7.6 Hz, 1H), 7.05 (d, *J*= 8.4 Hz, 2H), 6.72 (d, *J* = 7.6 Hz, 1H), 3.34 (d, *J* = 12.8 Hz, 1H), 3.16 (d, *J* = 12.8 Hz, 1H), 3.04 (s, 3H), 1.59 (s, 3H). ^13^C-NMR (CDCl_3_): δ 179.1, 146.8, 144.0, 142.4, 140.8, 136.0, 132.6, 130.7, 130.7 (overlapped), 128.9, 128.9 (overlapped), 127.2, 127.2 (overlapped), 126.8, 126.8 (overlapped), 122.8, 122.8 (overlapped), 121.8, 50.1, 44.2, 26.1, 23.3. HRMS (ESI^+^): calcd 373.1547 for C_23_H_21_N_2_O_3_ [M + H]^+^; found, 373.1539.

*1,3-Dimethyl-3-(4-nitrobenzyl)-5-(trifluoromethyl)indolin-2-one* (**3l**). ^1^H-NMR (CDCl_3_): δ 7.99 (d, *J* = 8.8 Hz, 2H), 7.49 (d, *J* = 8.0 Hz, 1H), 7.45 (s, 2H), 6.96 (d, *J* = 8.8 Hz, 2H), 6.69 (d, *J* = 8.0 Hz, 1H), 3.32 (d, *J* = 12.8 Hz, 1H), 3.11 (d, *J* = 12.8 Hz, 1H), 3.01 (s, 3H), 1.54 (s, 3H). ^13^C-NMR (CDCl_3_): δ 179.2, 147.0, 146.1, 143.4, 132.8, 130.5, 130.5 (overlapped), 126.2 (q, ^3^*J*_F-C_ = 4 Hz), 124.9 (q, ^2^*J*_F-C_ = 32 Hz), 124.4 (q, ^1^*J*_F-C_ = 270 Hz), 123.0,123.0 (overlapped), 120.2 (q, ^3^*J*_F-C_ = 4 Hz), 108.2, 50.1, 44.1, 26.3, 23.2. HRMS (ESI^+^): calcd 365.1108 for C_18_H_16_F_3_N_2_O_3_ [M + H]^+^; found, 365.1094.

*1,3,7-Trimethyl-3-(4-nitrobenzyl)indolin-2-one* (**3m**). ^1^H-NMR (CDCl_3_): δ 7.89, (d, *J* = 8.8 Hz, 2H), 7.05 (brd, *J* = 6.8 Hz, 1), 6.99–6.91 (m, 3H), 3.26 (d, *J* = 12.8 Hz, 1H), 3.24 (s, 3H), 3.04 (d, *J* = 12.8 Hz, 1H), 2.38 (s, 3H), 1.48 (s, 3H). ^13^C-NMR (CDCl_3_): δ 180.0, 146.9, 144.3, 140.9, 132.8, 132.1, 130.7, 130.7 (overlapped), 122.8, 122.8 (overlapped), 122.6, 121.0, 119.9, 49.5, 44.5, 29.4, 23.8, 19.0. HRMS (ESI^+^): calcd 311.1390 for C_18_H_19_N_2_O_3_ [M + H]^+^; found, 311.1377.

*7-Fluoro-1,3-dimethyl-3-(4-nitrobenzyl)indolin-2-one* (**3n**). ^1^H-NMR (CDCl_3_): δ 7.91 (d, *J* = 8.8 Hz, 2H), 7.01–6.89 (m, 5H), 3.28 (d, *J* = 13.2 Hz, 1H), 3.18 (s, 3H), 3.06 (d, *J* = 13.2 Hz, 1H), 1.51 (s, 3H). ^13^C-NMR (CDCl_3_): δ 178.9, 147.7 (d, ^1^*J*_F-C_ = 243 Hz), 147.0, 143.8, 135.2, 130.6, 130.6 (overlapped), 128.2, 123.2(d, ^3^*J*_F-C_ = 6.2 Hz), 123.0, 123.0 (overlapped), 118.9 (^3^*J*_F-C_ = 3.3 Hz), 116.4 (^2^*J*_F-C_ = 19.4Hz), 50.5, 44.3, 29.8, 23.6. HRMS (ESI^+^): calcd 315.1139 for C_17_H_16_FN_2_O_3_ [M + H]^+^; found, 315.1127.

*1-Methyl-1-(4-nitrobenzyl)-5,6-dihydro-1H-pyrrolo[3,2,1-ij]quinolin-2(4H)-one* (**3o**). ^1^H-NMR (CDCl_3_): δ 7.88–7.84 (m, 2H), 7.01–6.99 (m, 3H), 6.93–6.92 (m, 2H), 3.56–3.47 (m, 1H), 3.40–3.33 (m, 1H), 3.23 (d, *J* = 12.2 Hz, 1H), 3.07 (d, *J* = 12.2 Hz, 1H), 2.15-2.13 (m, 3H), 1.84–1.81 (m, 2H), 1.50 (s, 3H). ^13^C-NMR (CDCl_3_): δ 177.8, 146.6, 144.3, 138.7, 130.6, 130.6 (overlapped), 130.5, 127.0, 122.5, 122.5 (overlapped), 121.9, 120.8, 120.2, 51.2, 44.0, 38.6, 24.3, 22.7, 21.0. HRMS (ESI^+^): calcd 323.1390 for C_19_H_19_N_2_O_3_ [M + H]^+^; found, 323.1402.

*1-Methyl-3-(4-nitrobenzyl)-3-phenylindolin-2-one* (**3p**). ^1^H-NMR (CDCl_3_): δ 7.90 (d, *J* = 8.4 Hz, 2H), 7.50 (d, *J* = 8.0 Hz, 2H), 7.40–7.27 (m, 5H), 7.14 (t, *J* = 7.6 Hz, 1H), 7.04 (d, *J* = 8.0 Hz, 2H), 6.67 (d, *J* = 7.6, Hz, 1H), 3.86 (d, *J* = 12.4 Hz, 1H), 3.55 (d, *J* = 12.4 Hz, 1H), 2.99 (s, 3H). ^13^C-NMR (CDCl_3_): δ 177.2, 146.8, 143.6, 143.5, 139.1, 130.9, 130.9 (overlapped), 130.4, 129.0, 128.8, 128.8 (overlapped), 127.8, 127.1, 127.1 (overlapped), 125.3, 122.6, 122.5 122.5 (overlapped), 108.4, 57.9, 43.5, 26.2. HRMS (ESI^+^): calcd 359.1390 for C_22_H_19_N_2_O_3_ [M + H]^+^; found,359.1382.

*1-Methyl-3-(3-nitrobenzyl)-3-phenylindolin-2-one* (**3r**). ^1^H-NMR (CDCl_3_): δ 7.95-7.92 (m, 1H) 7.64 (s, 1H), 7.25–7.19 (m, 4H), 7.10 (t, *J* = 7.2 Hz, 1H), 6.62 (d, *J* = 8.0 Hz, 1H), 3.30 (d, *J* = 13.2 Hz, 1H), 3.10 (d, *J* = 13.2 Hz, 1H), 2.99 (s, 3H), 1.53 (s, 3H). ^13^C-NMR (CDCl_3_): δ 179.2, 147.5, 142.9, 138.3, 135.9, 131.9, 128.4, 128.3, 124.4, 123.0, 122.6, 121.6, 108.1, 49.8, 44.0, 25.9, 22.9. HRMS (ESI^+^): calcd 297.1234 for C_17_H_17_N_2_O_3_ [M + H]^+^; found,297.1240.

## 4. Conclusions

In summary, we report an ascorbic acid-initiated radical tandem cyclization of *N*-arylacrylamides with 4-nitroaniline to produce 3,3-disubstituted oxindoles. This newly developed method avoids the use of excess oxidant and metal catalyst and meets the demands of the environmental friendly trend in organic synthesis. Furthermore, the reaction can proceed smoothly without light irradiation at room temperature. However, there is an obvious limitation for the reaction in that the scope of aniline susbtrates seems limited to 4-nitroaniline. Further optimization of the reaction conditions is still underway in our lab to broaden the scope of applicable anilines.

## Supplementary Materials

Supplementary materials can be accessed at: http://www.mdpi.com/1420-3049/20/09/15631/s1.
