# Supplementary material for: Ascorbic Acid-Initiated Tandem Radical Cyclization of N-Arylacrylamides to Give 3,3-Disubstituted Oxindoles
Source: Molecules. 2015 Aug 27;20(9):15631–42. doi: 10.3390/molecules200915631 (PMC6332015; doi:10.3390/molecules200915631)
Supplement: Supplementary file 1 [file molecules-20-15631-s001.pdf]

Supplementary Information

Table of Contents

1. HPLC-ESI-HRMS analysis of crude products **3m** S1

2. ^{1}H-NMR and ^{13}C-NMR spectroscopy of compounds **3** S3

1. HPLC-ESI-HRMS Analysis of Crude Tandem Cyclization Products with *ortho*-Substituted *N*-Aryacrylamide as Substrate

Significantly decreased yields of the desired compounds were observed when the *ortho*-substituted *N*-arylacrylamides were used as reaction substrates. The reaction mixture was much more complex. In these *ortho*-substituted cases, α-hydroxyl amide derivative (**A**) and simple C-C double bond Meerwein radical addition products (**C**) were detected as major byproducts. Due to the steric effect, the intermolecular cyclization was not favored, thus, radical intermediate **11** (Scheme 4, in the manuscript) could be oxidized by trace oxygen in the reaction system to provide byproduct A or quenched through an H-atom abstraction process to afford byproduct **C** (Scheme S1).

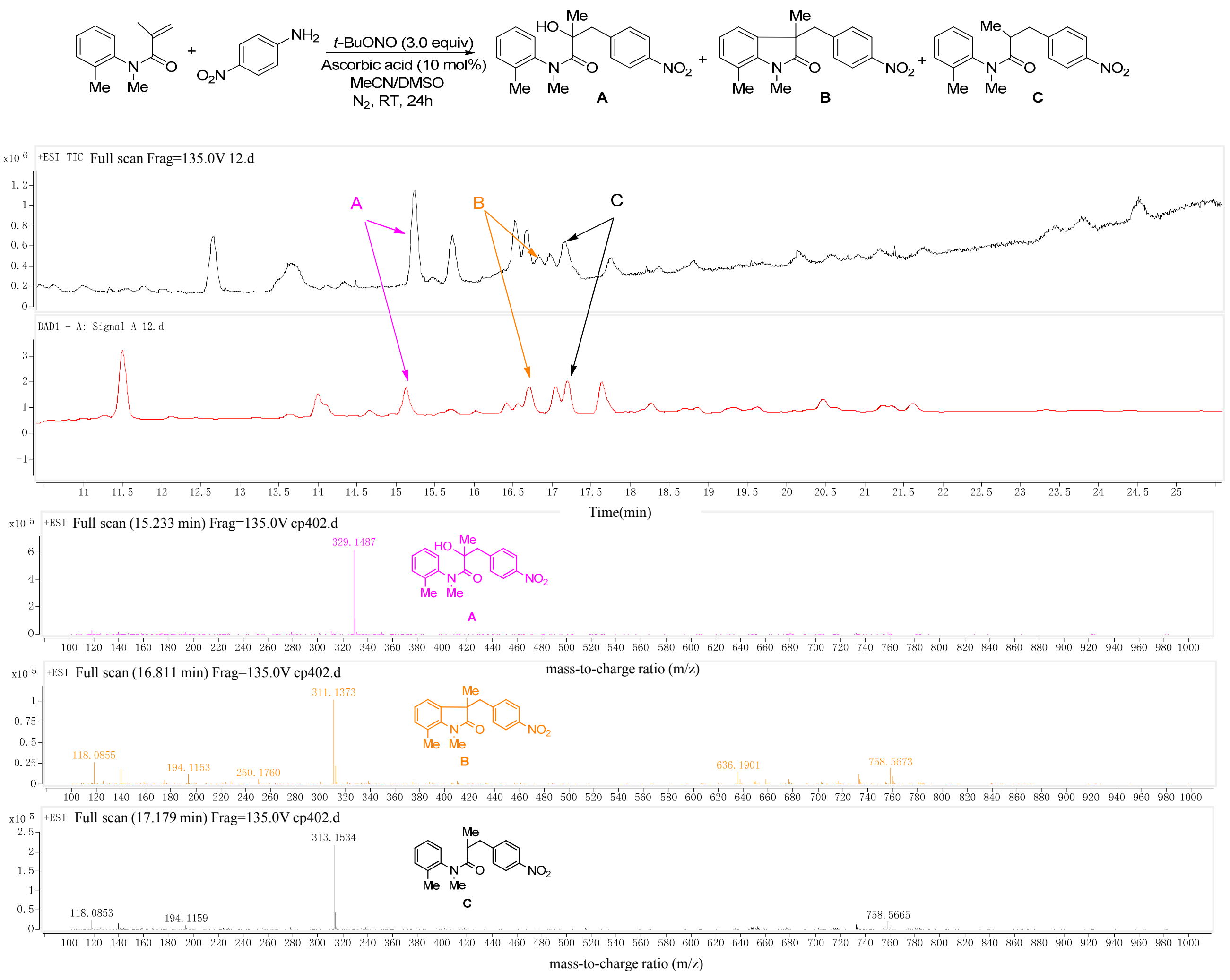

Scheme S1. HPLC-ESI-HRMS analysis of crude product.

Because byproduct **C** is a typical Meerwein radical addition product, our work mainly focused on the structure identification of byproduct A. We tried to isolate the byproduct **A** by silicon chromatography. However, the reaction product was complex and we didn't obtain pure compounds **A**. Thus, we performed the MS/MS analysis of the crude product and hoped to confirm the structure of compound **A** based on the MS/MS fragmentation behavior. As shown in Scheme 5, the MS/MS spectra of byproduct A showed a characteristic ion $[M + H - 18]^+$ at 311.1411 corresponding to the loss a H_2O molecule from the cation $[M + H]^+$ at 329.1487. Based on the proposed reaction mechanism, a hydroxyl group should exist in compound A.

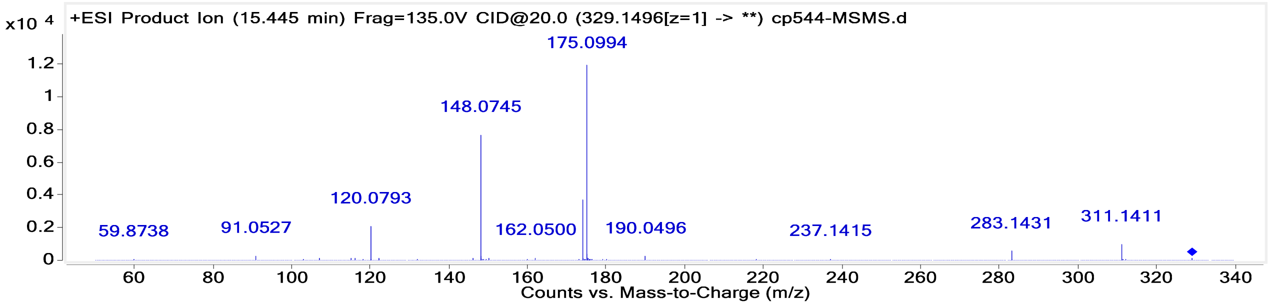

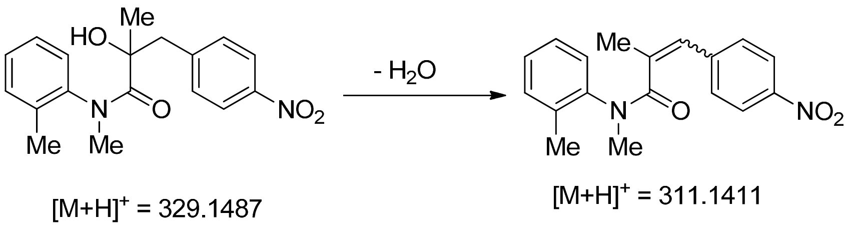

Scheme S2. MS/MS fragmentation pathway of byproduct **A**.

2. NMR Spectra of Compounds 3

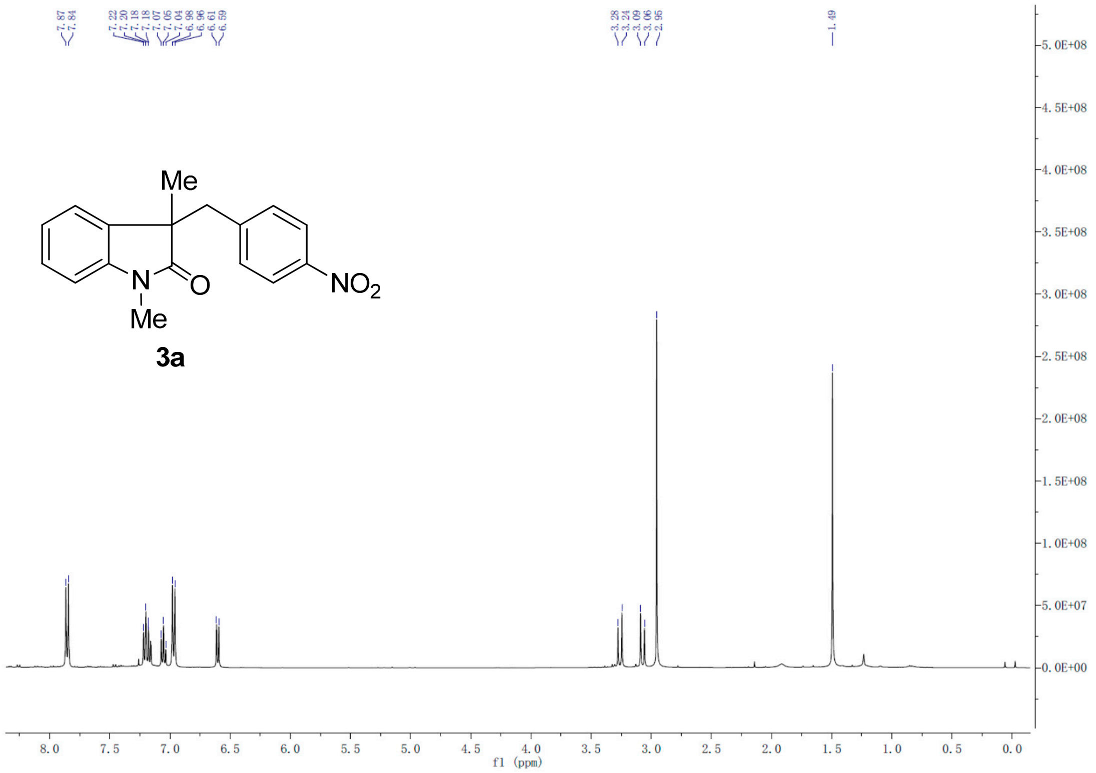

Figure S1. ^{1}H-NMR spectra of compound **3a**.

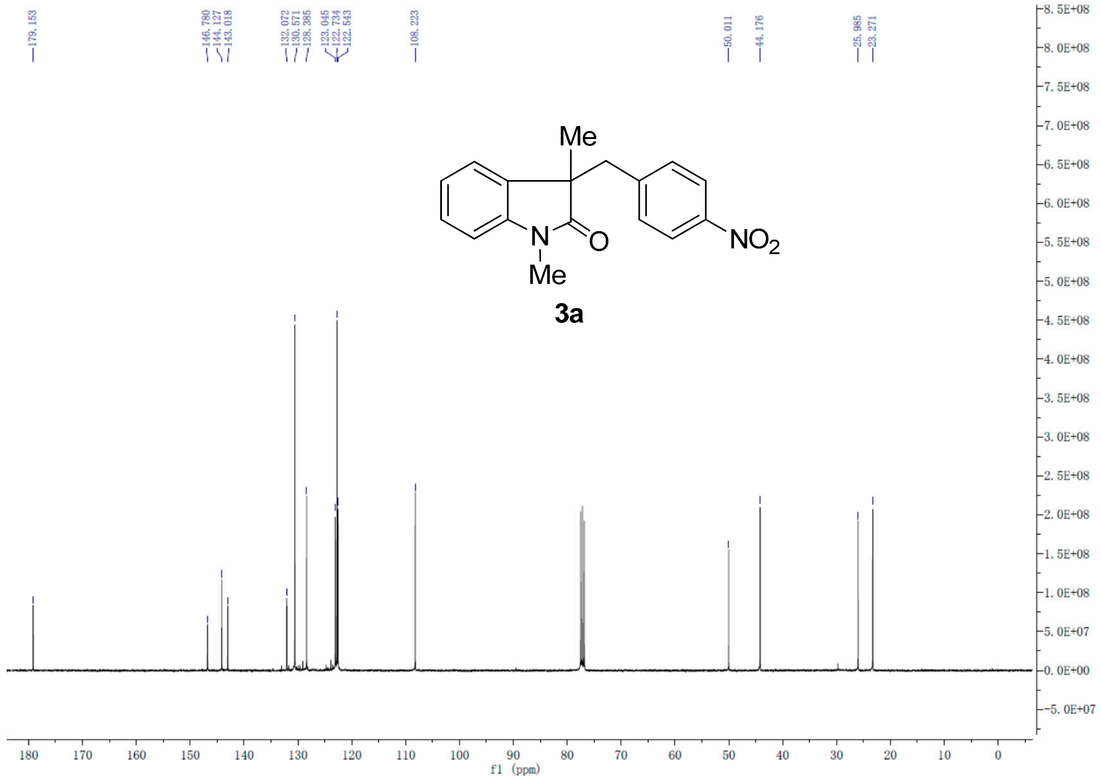

Figure S2. ^{13}C-NMR spectra of compound **3a**.

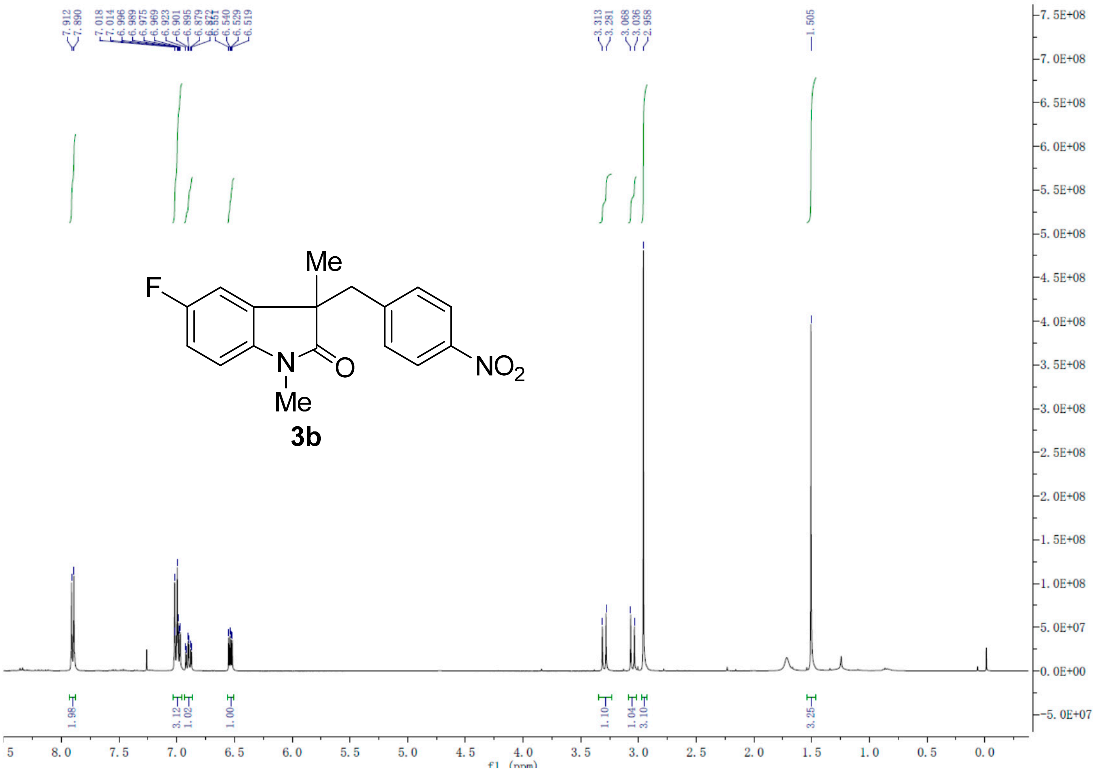

Figure S3. ^{1}H-NMR spectra of compound **3b**.

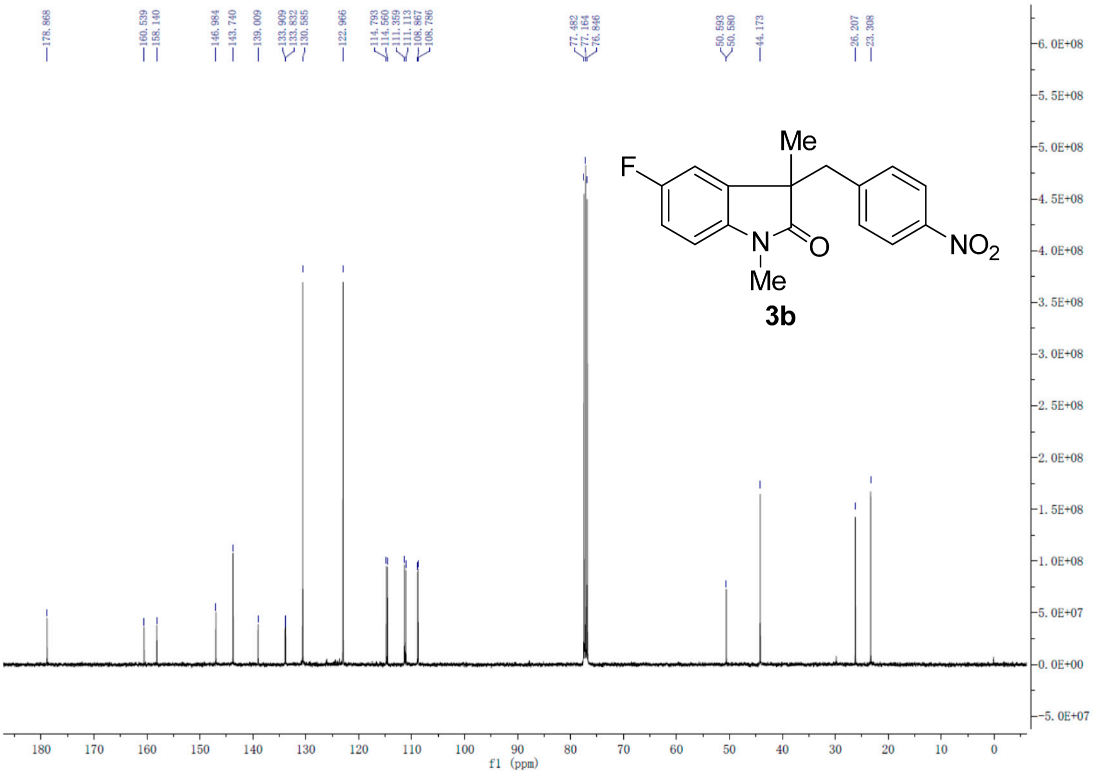

Figure S4. ^{13}C-NMR spectra of compound **3b**.

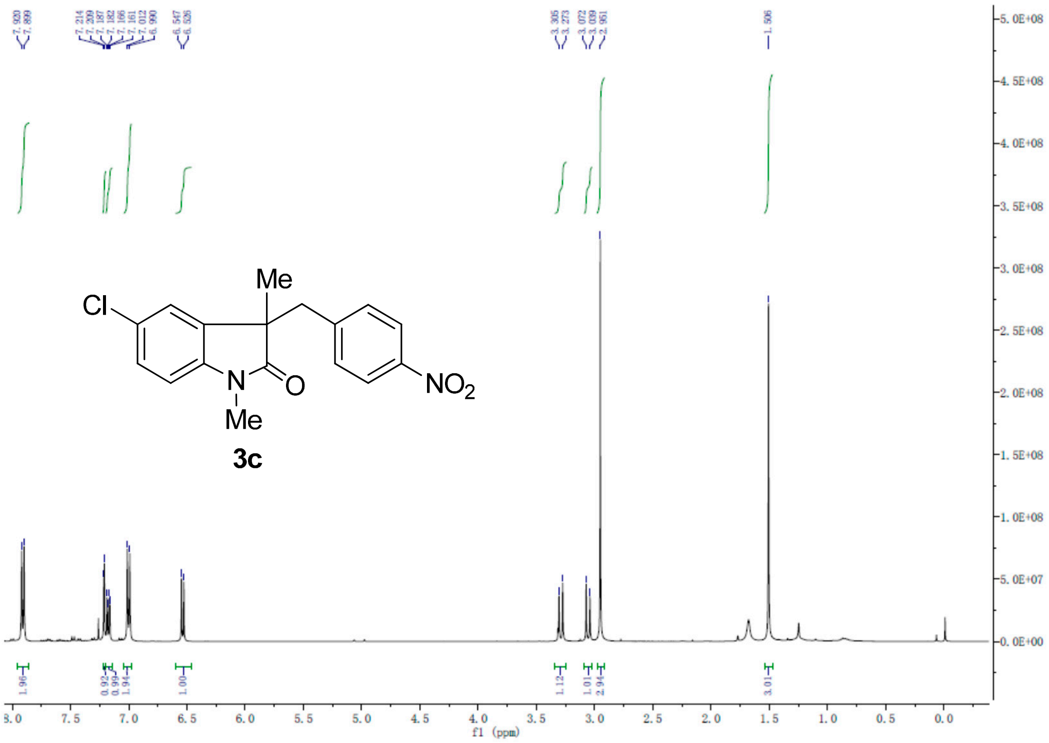

Figure S5. ^{1}H-NMR spectra of compound **3c**.

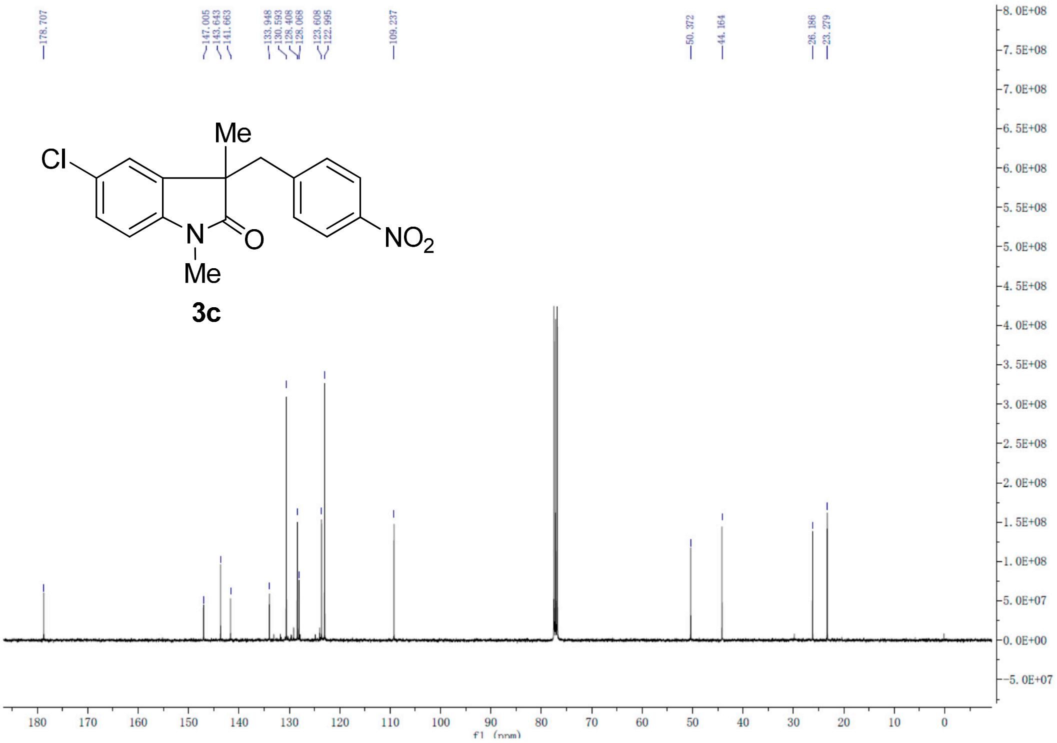

Figure S6. ^{13}C-NMR spectra of compound **3c**.

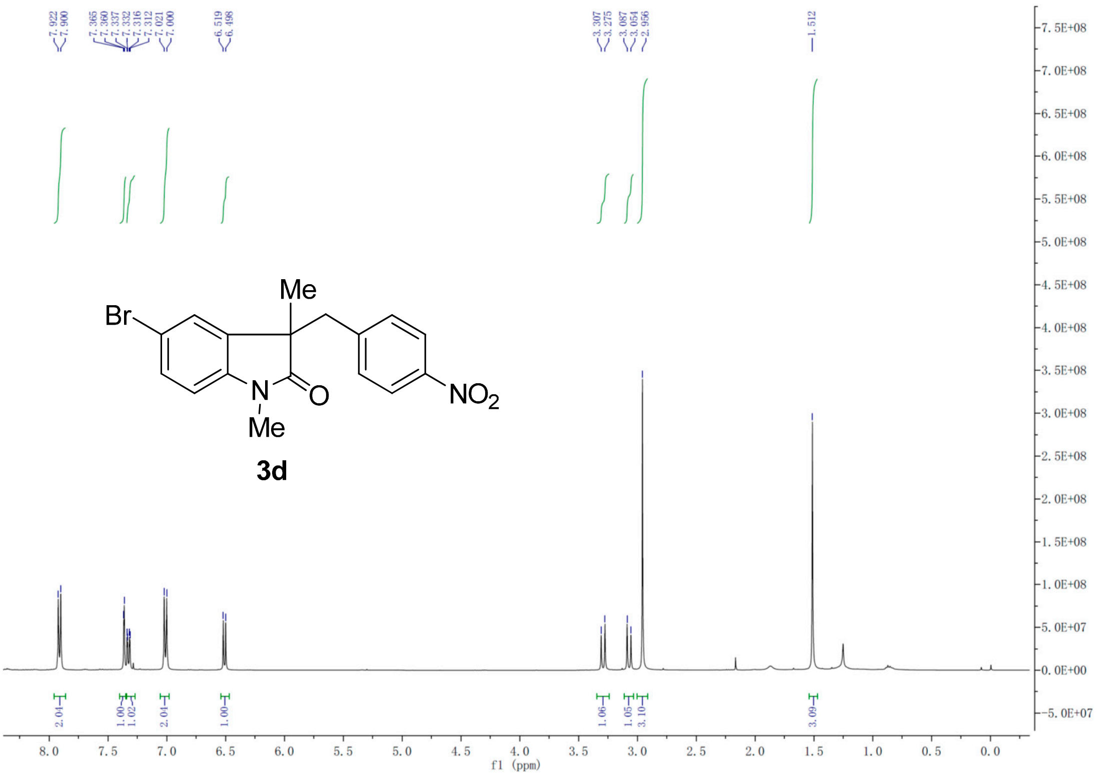

Figure S7. ^{1}H-NMR spectra of compound **3d**.

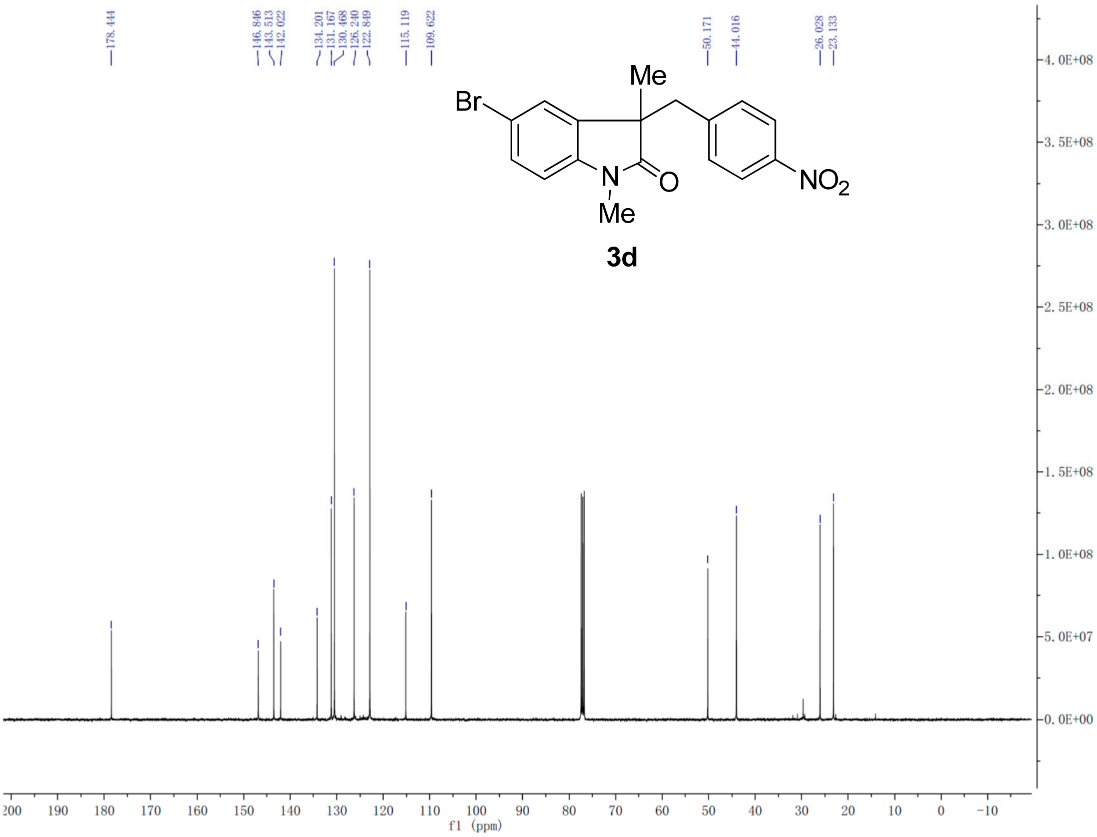

Figure S8. ^{13}C-NMR spectra of compound **3d**.

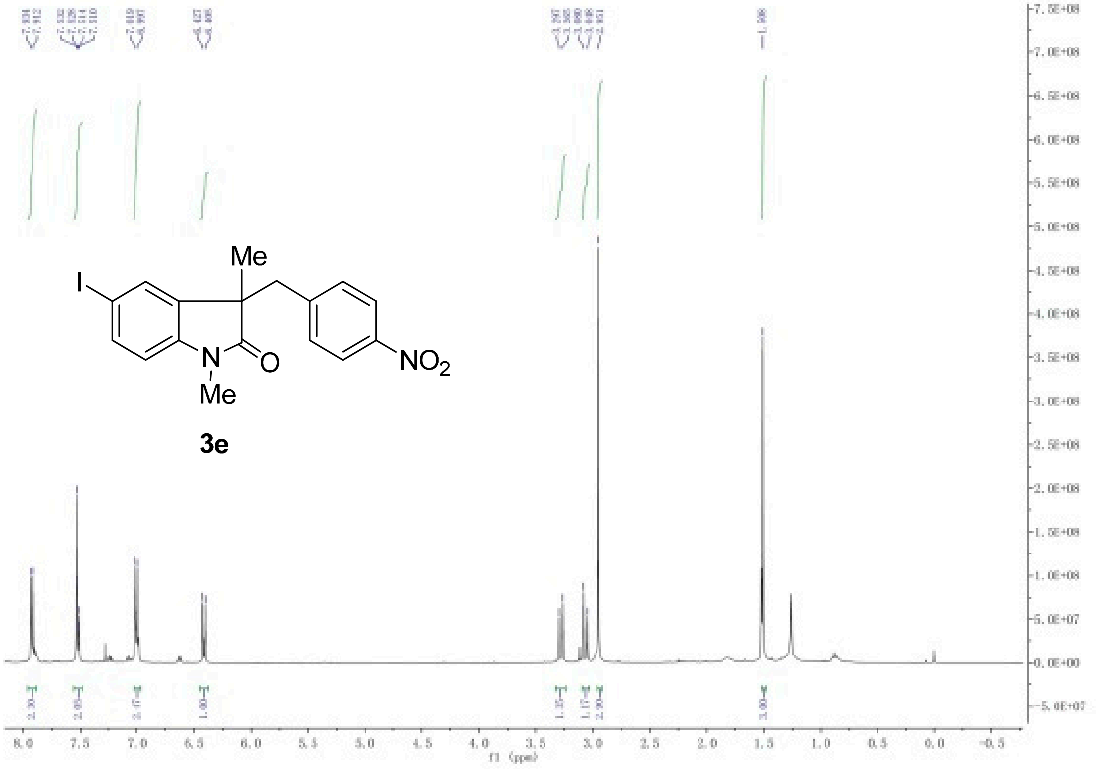

Figure S9. ^{1}H-NMR spectra of compound **3e**.

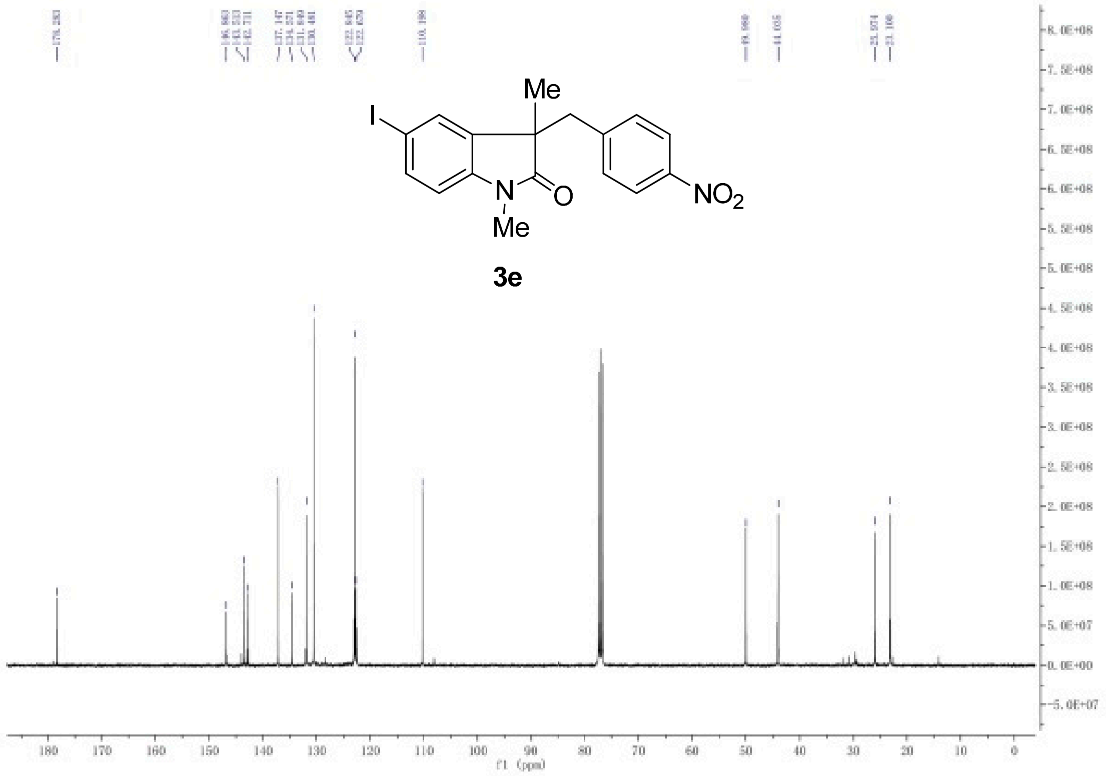

Figure S10. ^{13}C-NMR spectra of compound **3e**.

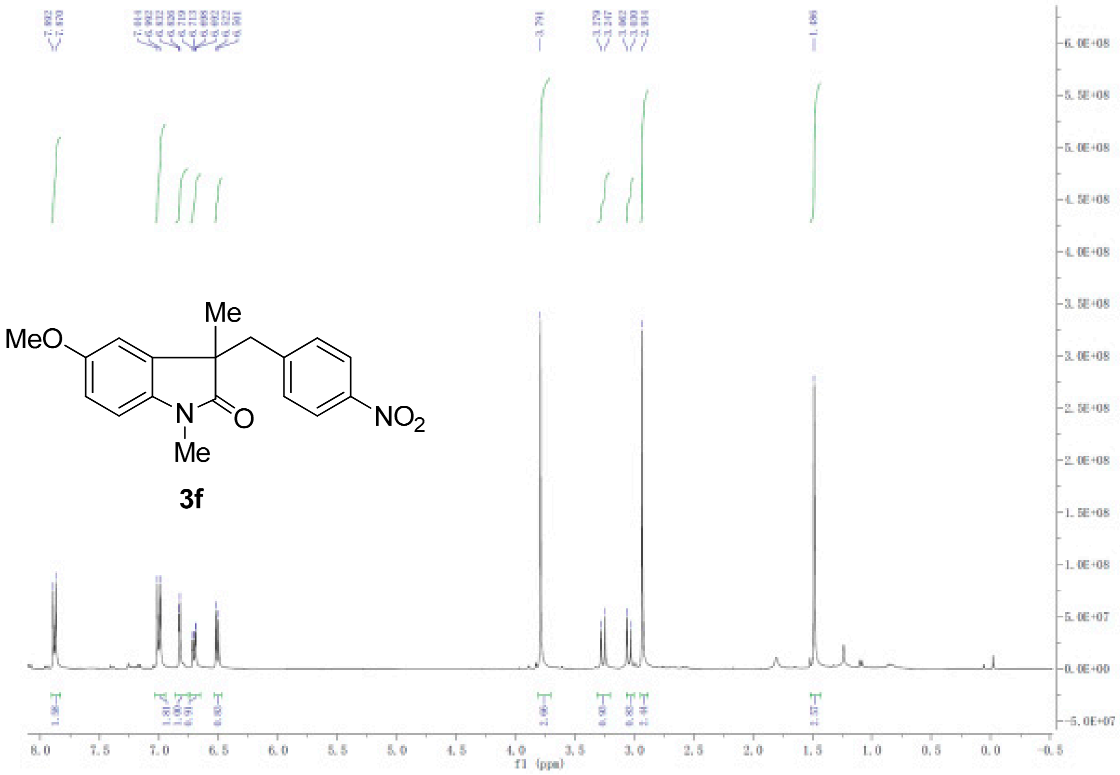

Figure S11. ^{1}H-NMR spectra of compound **3f**.

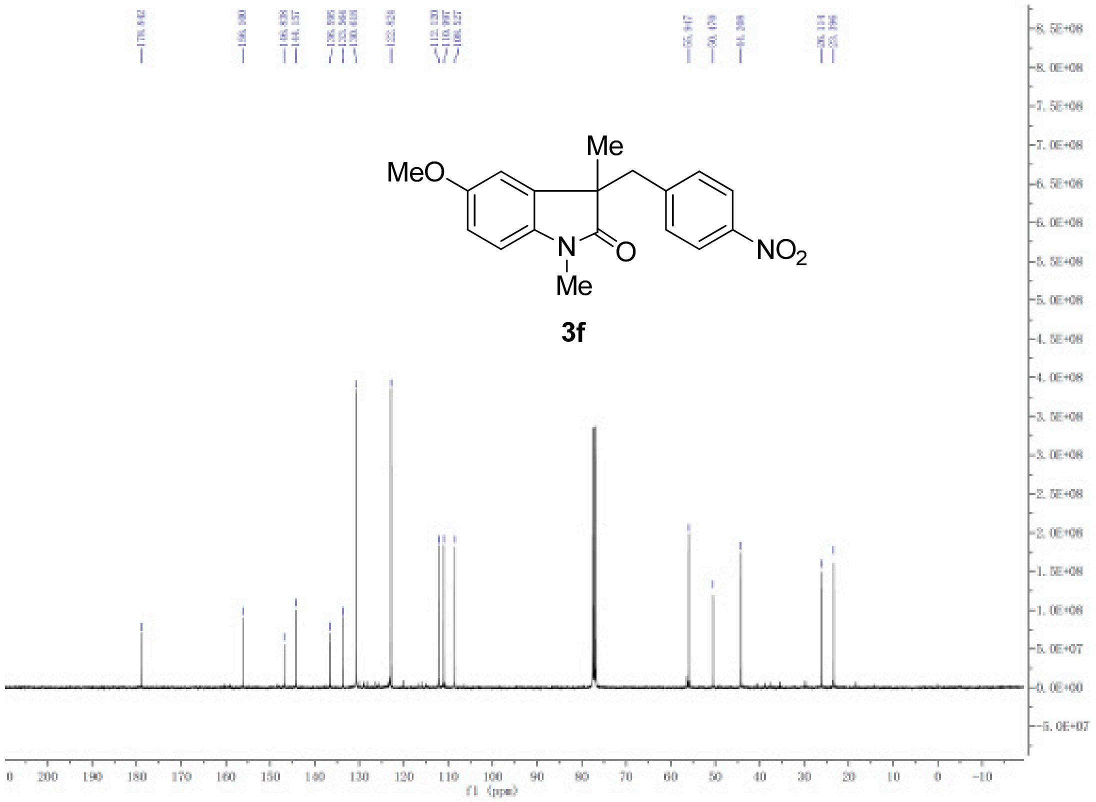

Figure S12. ^{13}C-NMR spectra of compound **3f**.

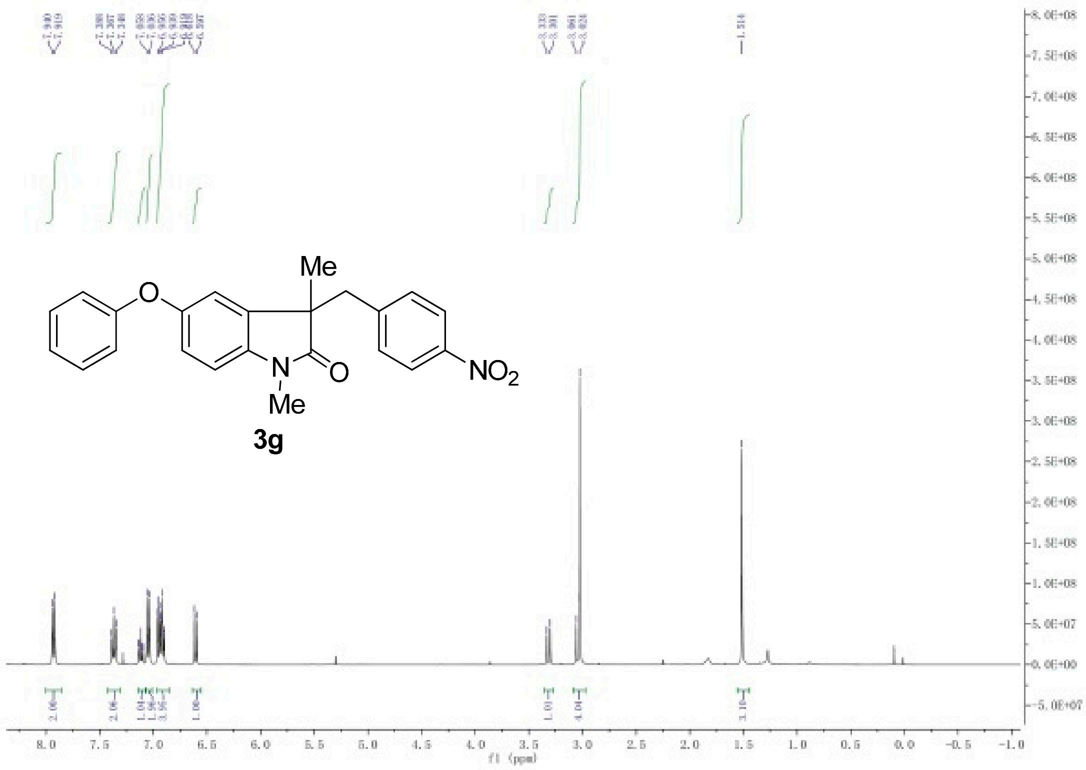

Figure S13. ^{1}H-NMR spectra of compound **3g**.

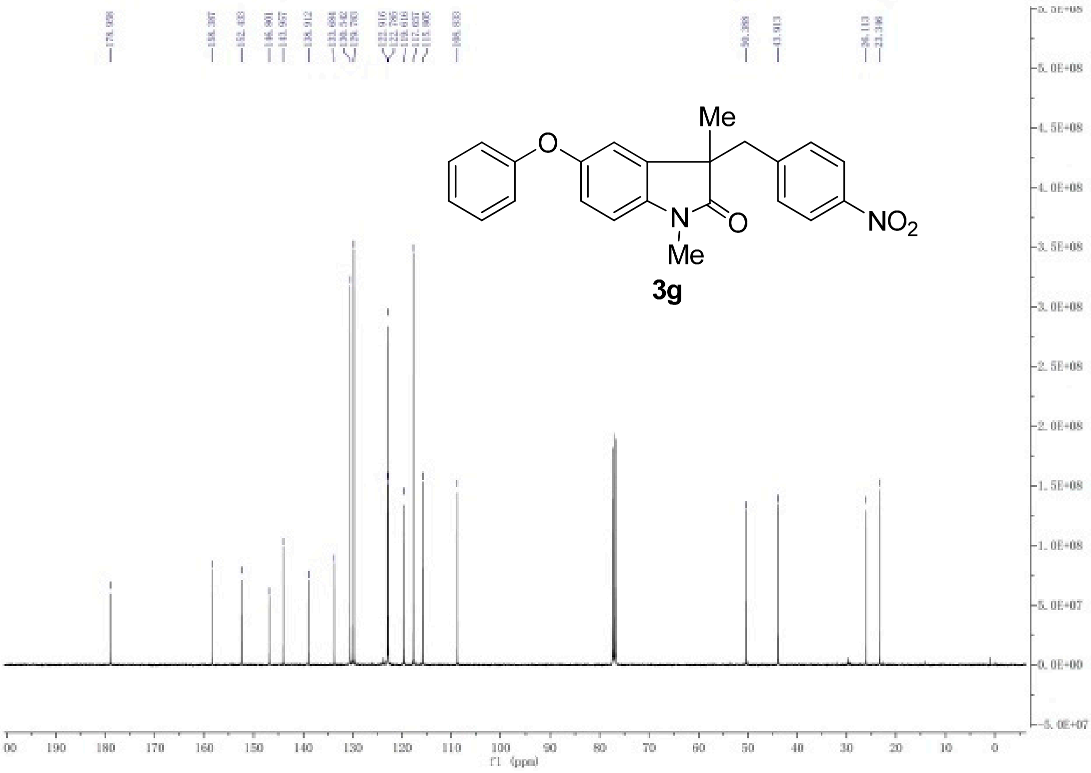

Figure S14. ^{13}C-NMR spectra of compound **3g**.

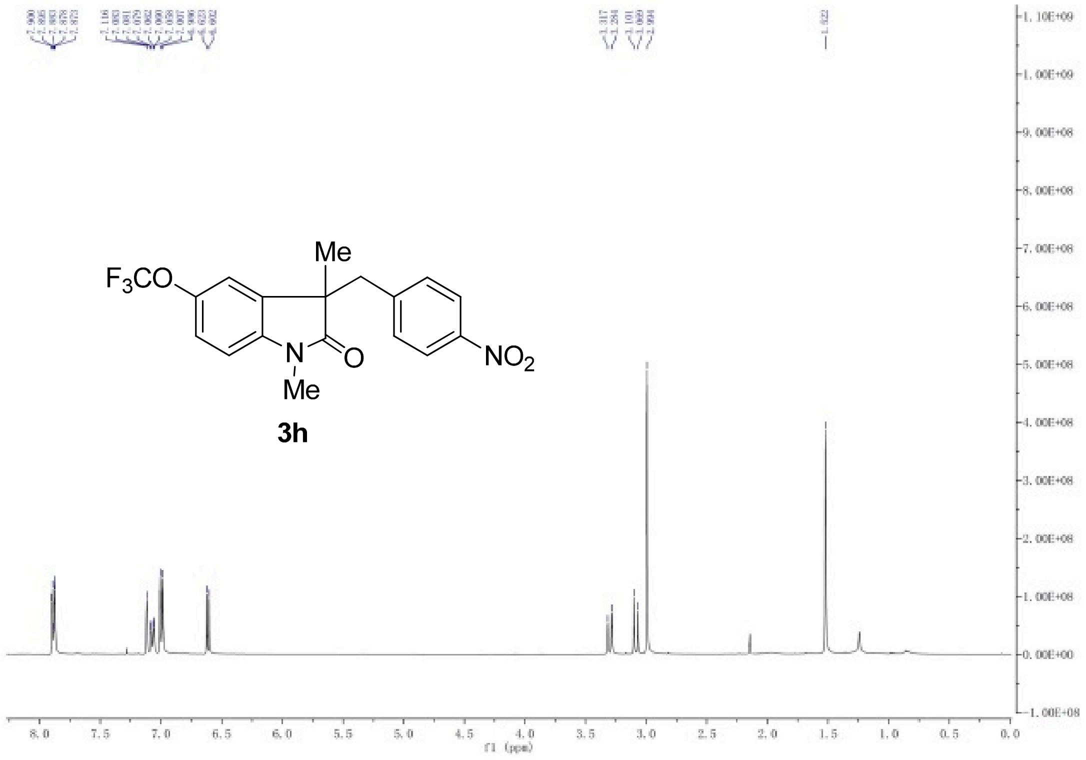

Figure S15. ^{1}H-NMR spectra of compound **3h**.

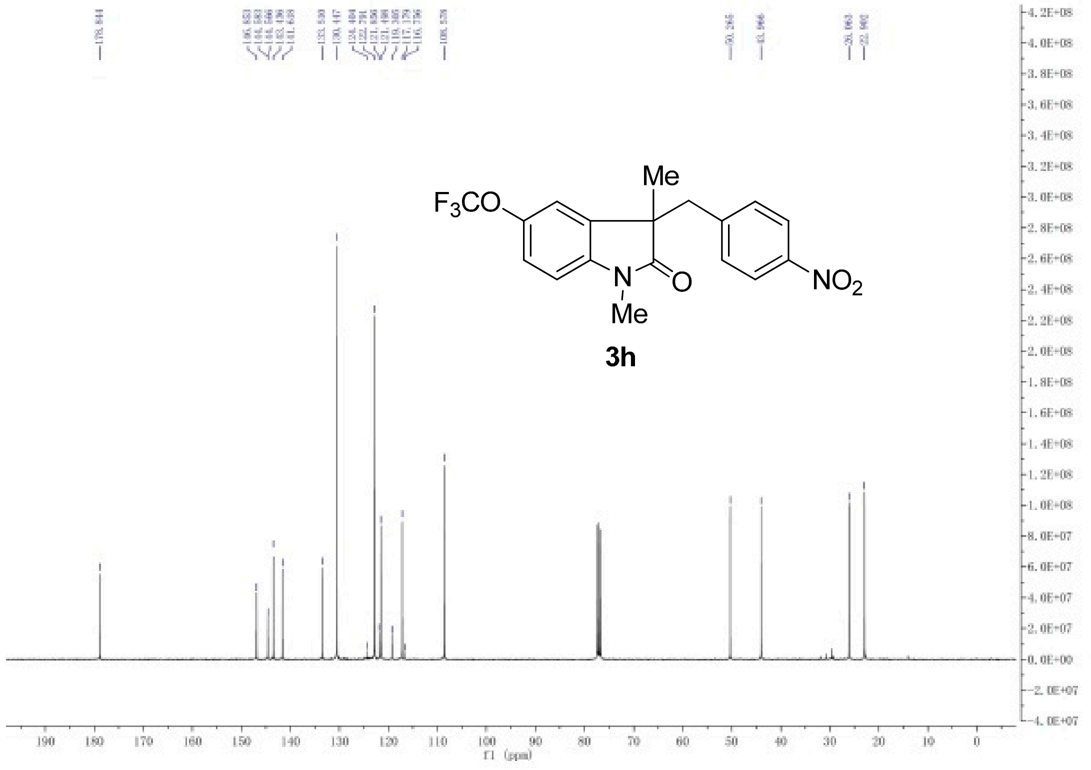

Figure S16. ^{13}C-NMR spectra of compound **3h**.

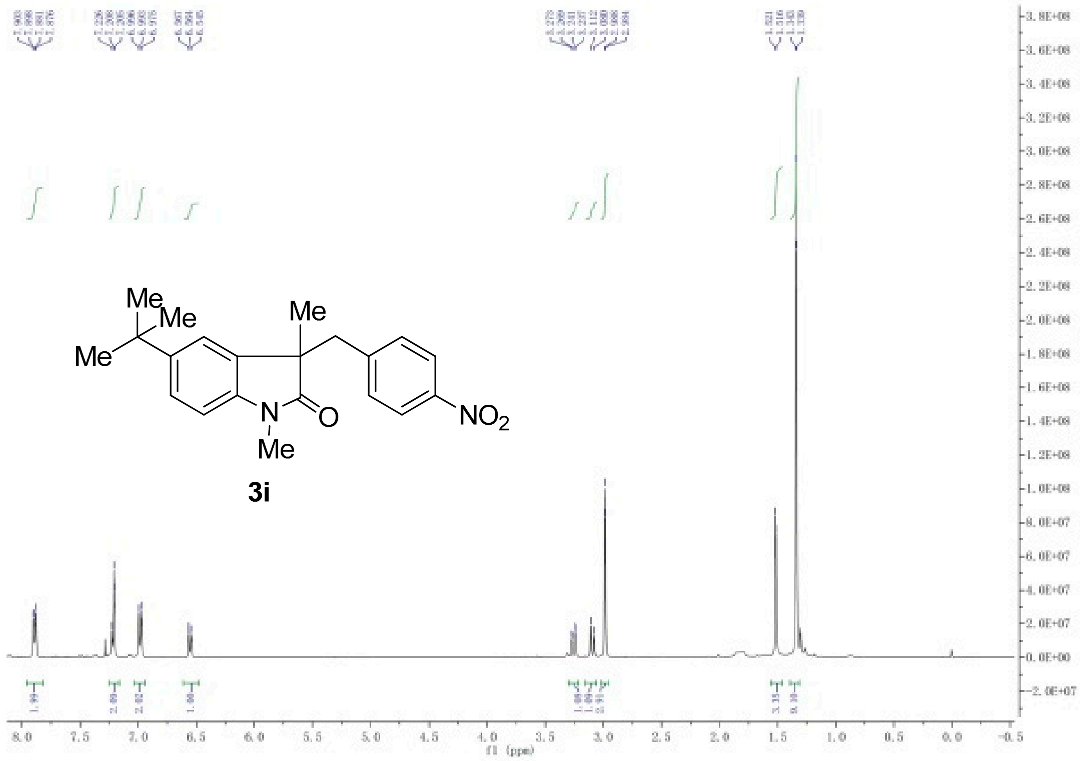

Figure S17. ^{1}H-NMR spectra of compound **3i**.

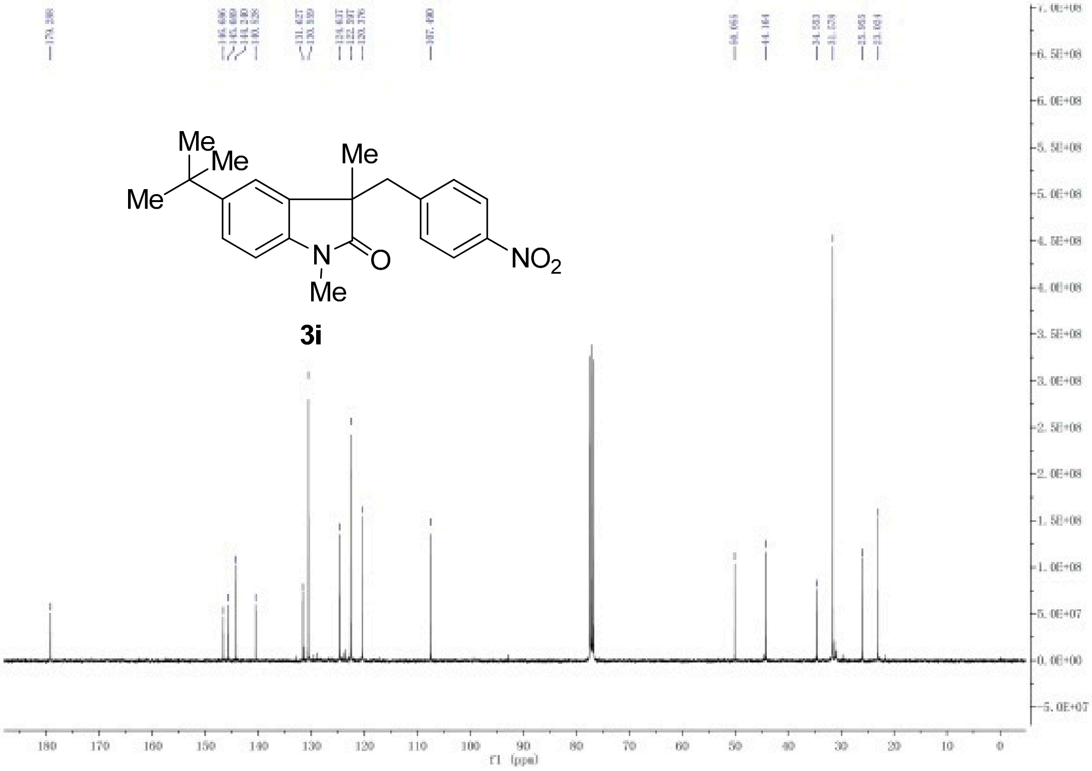

Figure S18. ^{13}C-NMR spectra of compound **3i**.

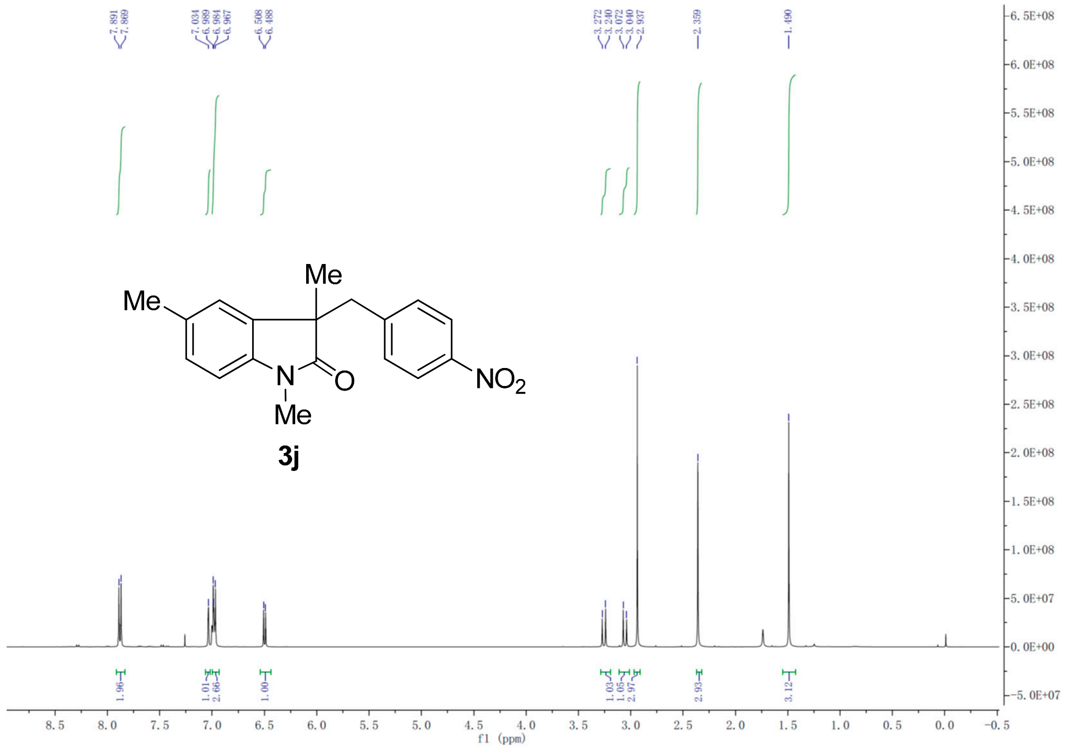

Figure S19. ^{1}H-NMR spectra of compound **3j**.

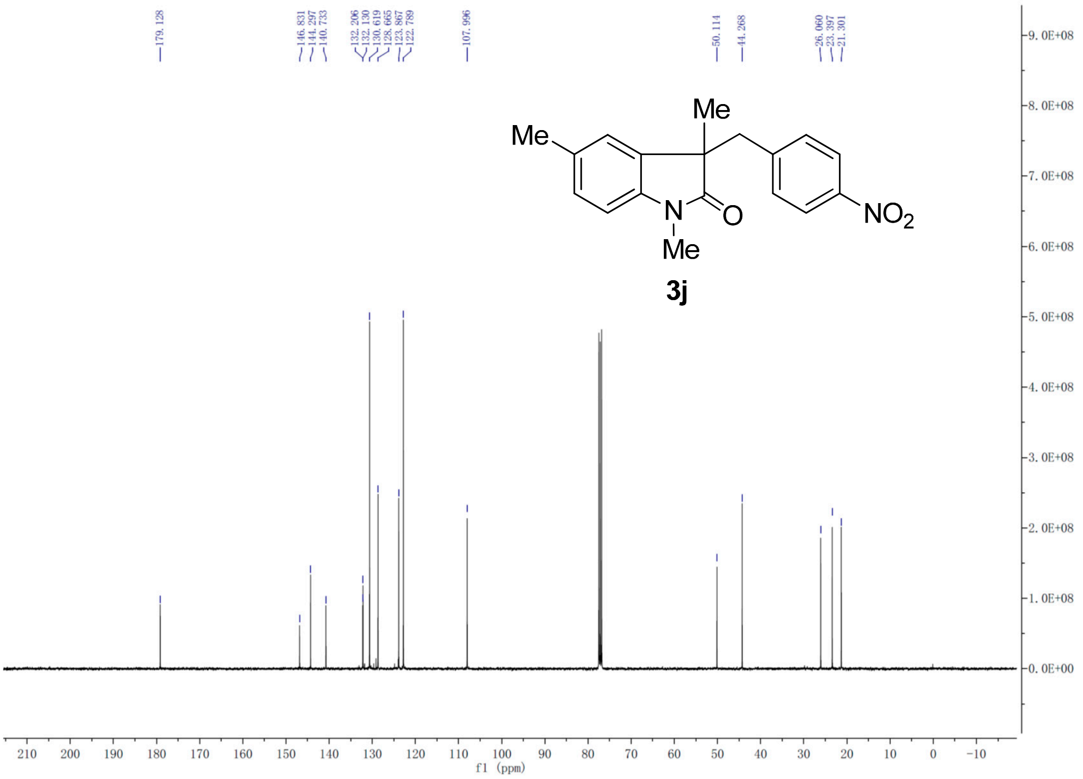

Figure S20. ^{13}C-NMR spectra of compound **3j**.

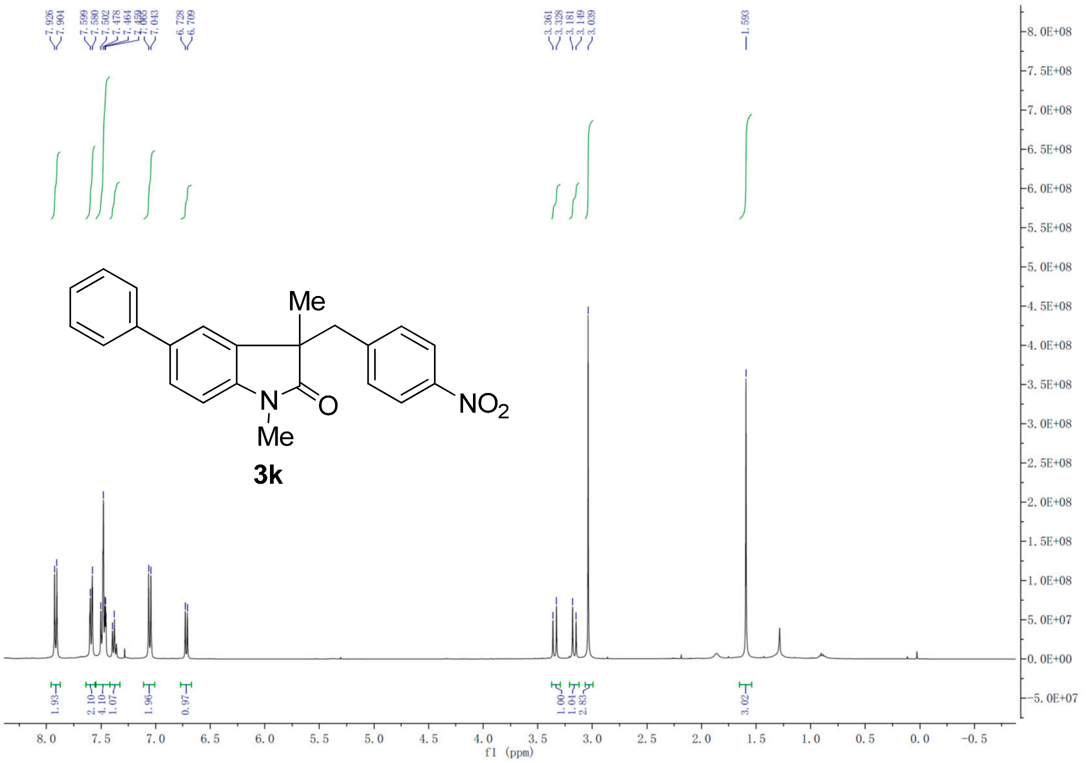

Figure S21. ^{1}H-NMR spectra of compound **3k**.

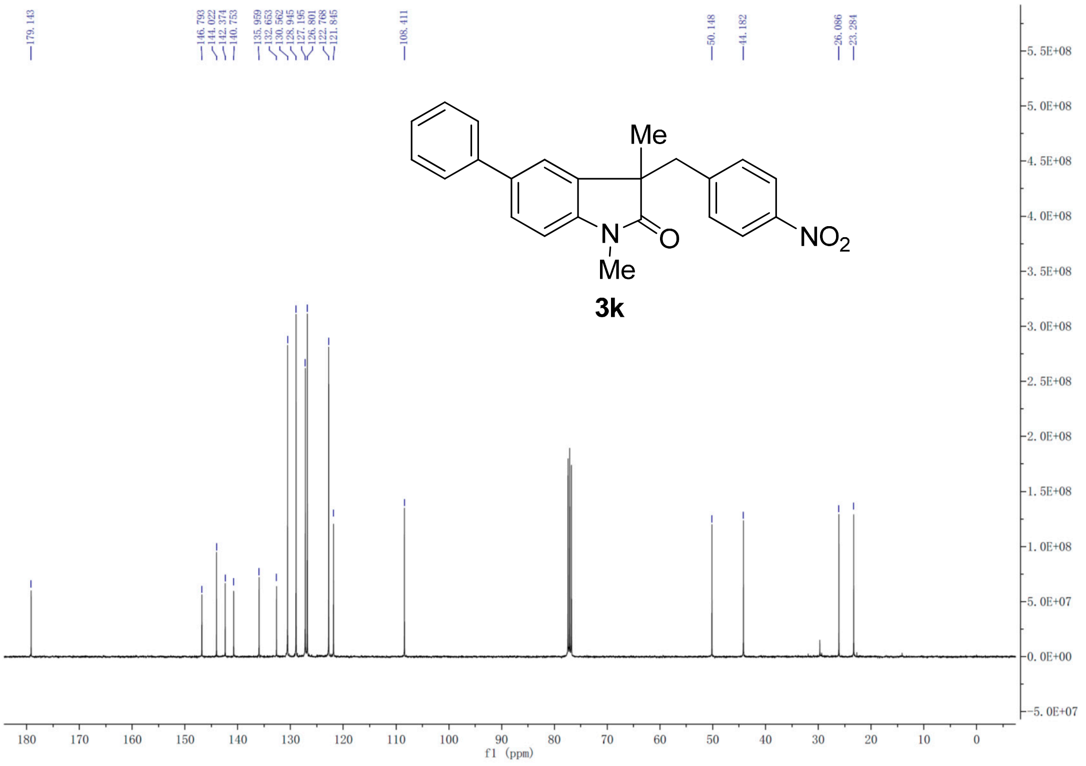

Figure S22. ^{13}C-NMR spectra of compound **3k**.

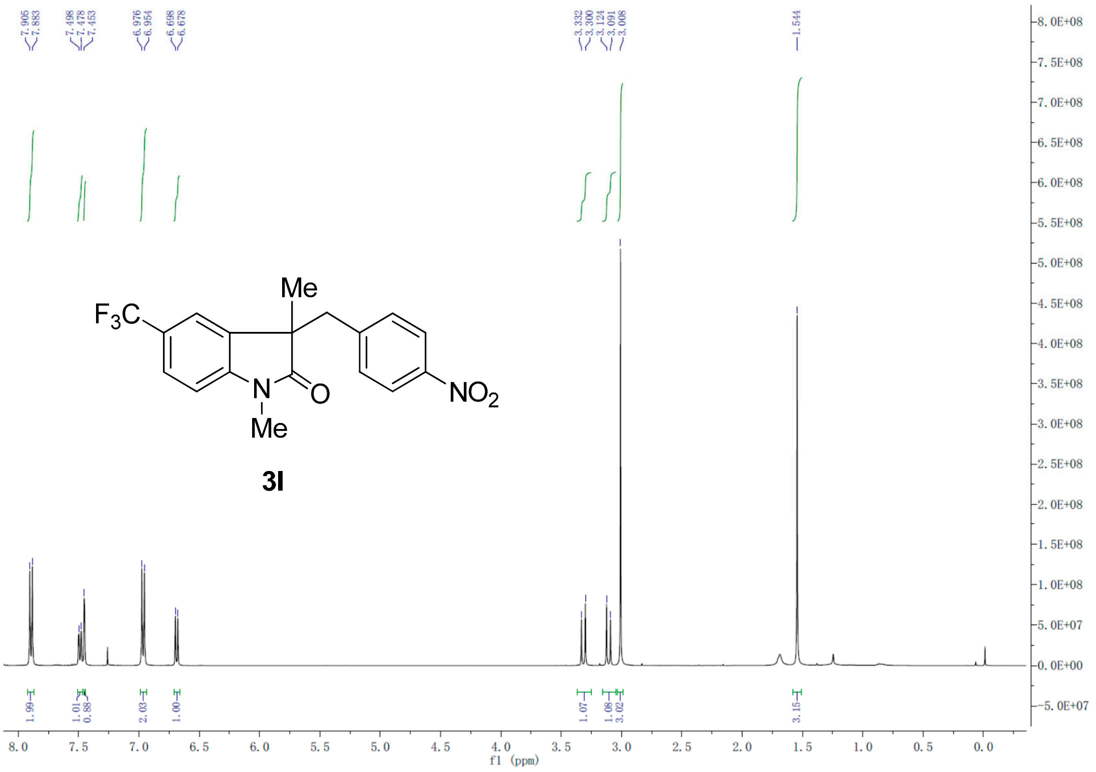

Figure S23. ^{1}H-NMR spectra of compound **3l**.

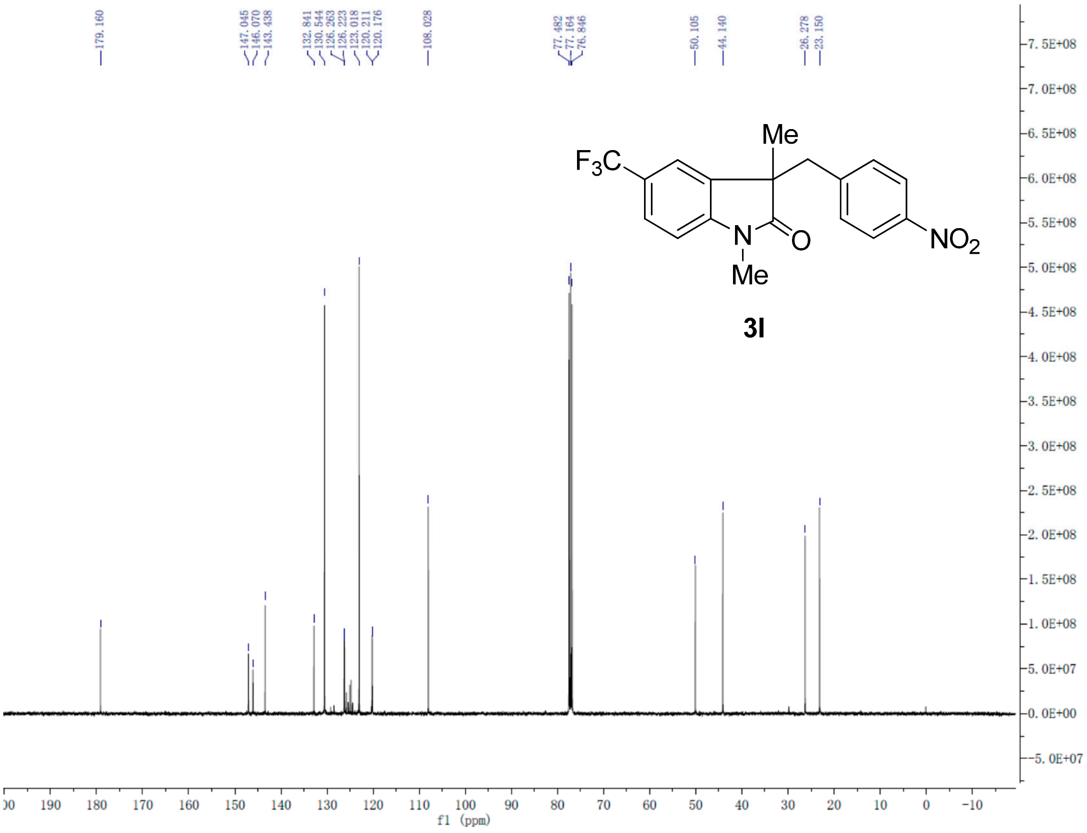

Figure S24. ^{13}C-NMR spectra of compound **3l**.

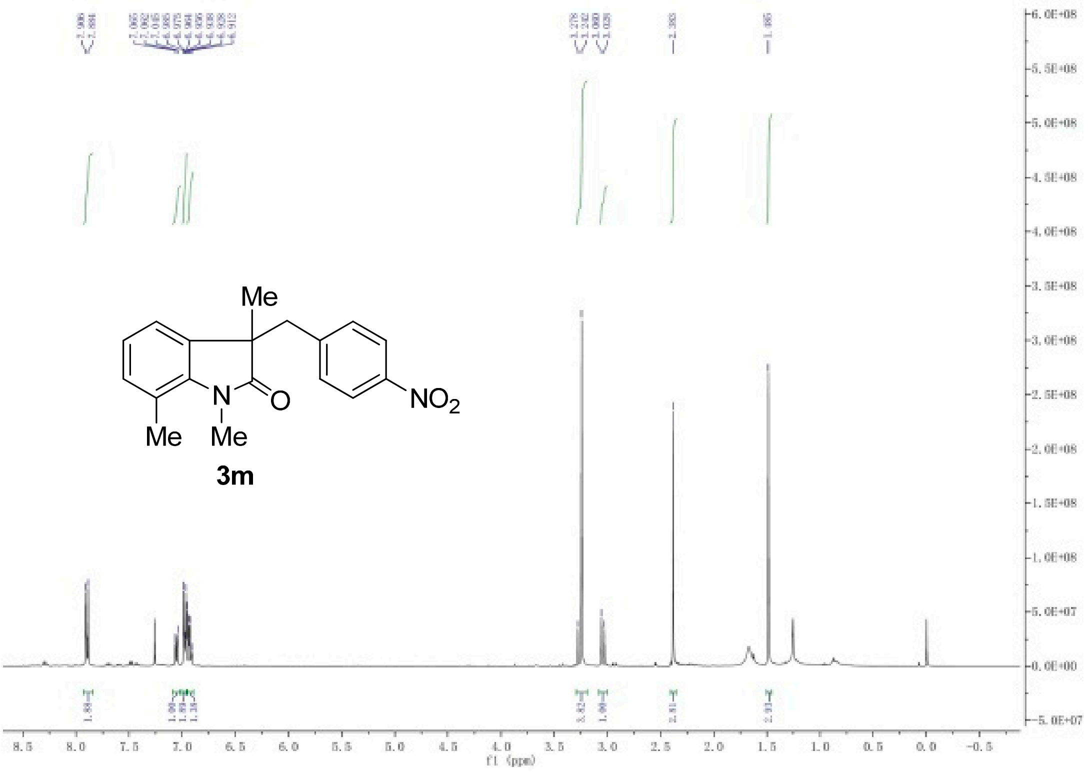

Figure S25. ^{1}H-NMR spectra of compound **3m**.

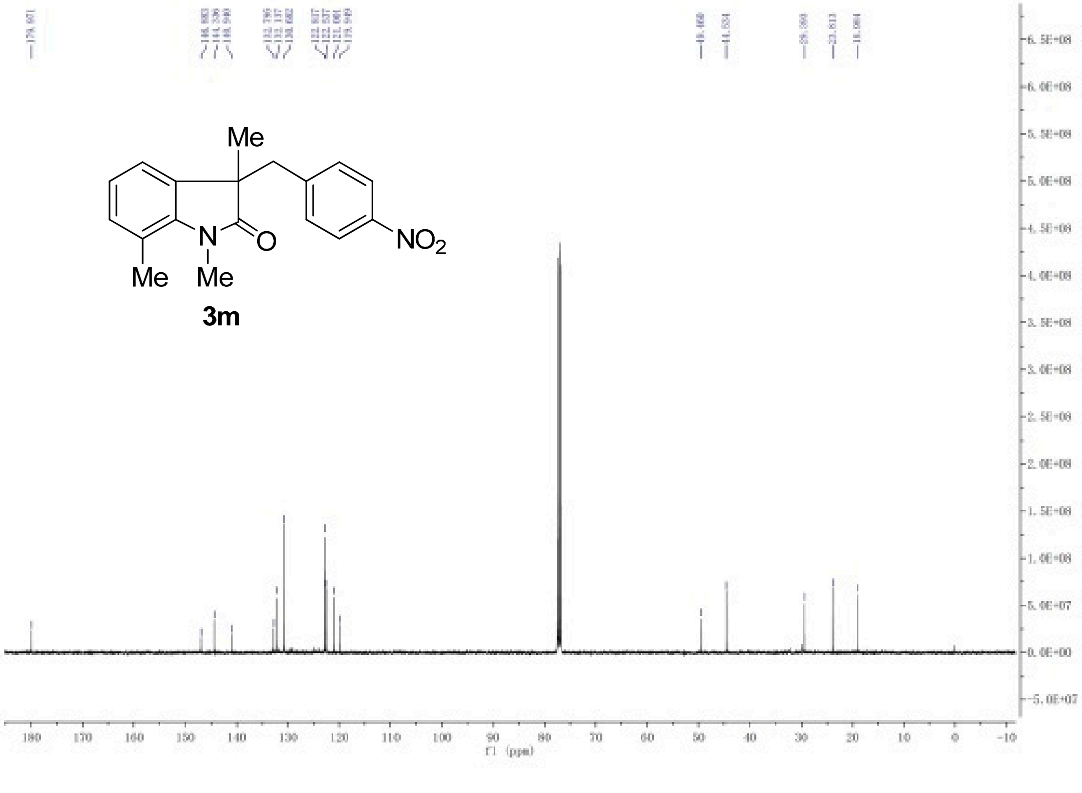

Figure S26. ^{13}C-NMR spectra of compound **3m**.

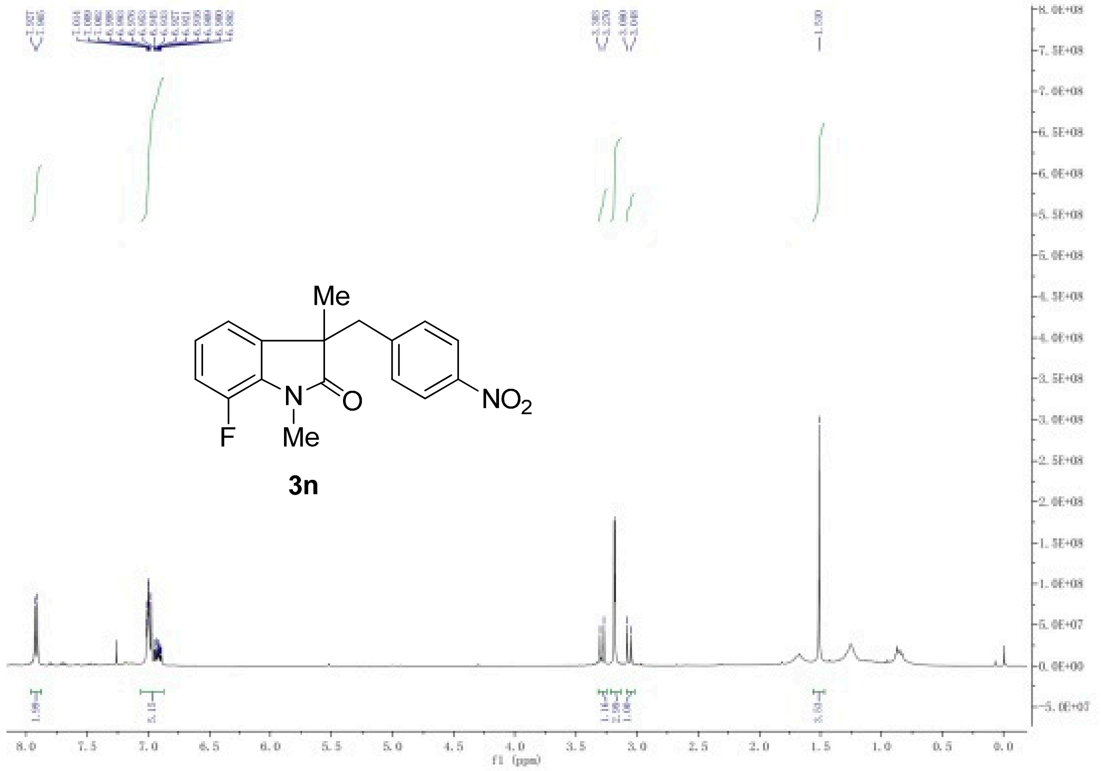

Figure S27. ^{1}H-NMR spectra of compound **3n**.

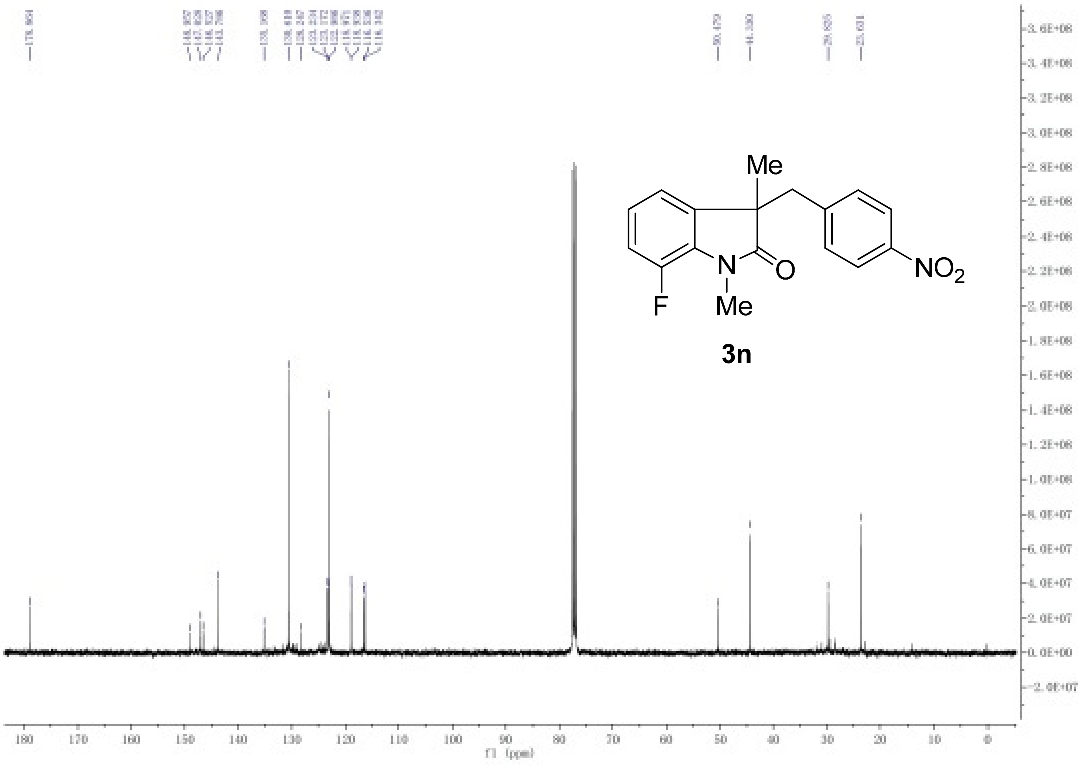

Figure S28. ^{13}C-NMR spectra of compound **3n**.

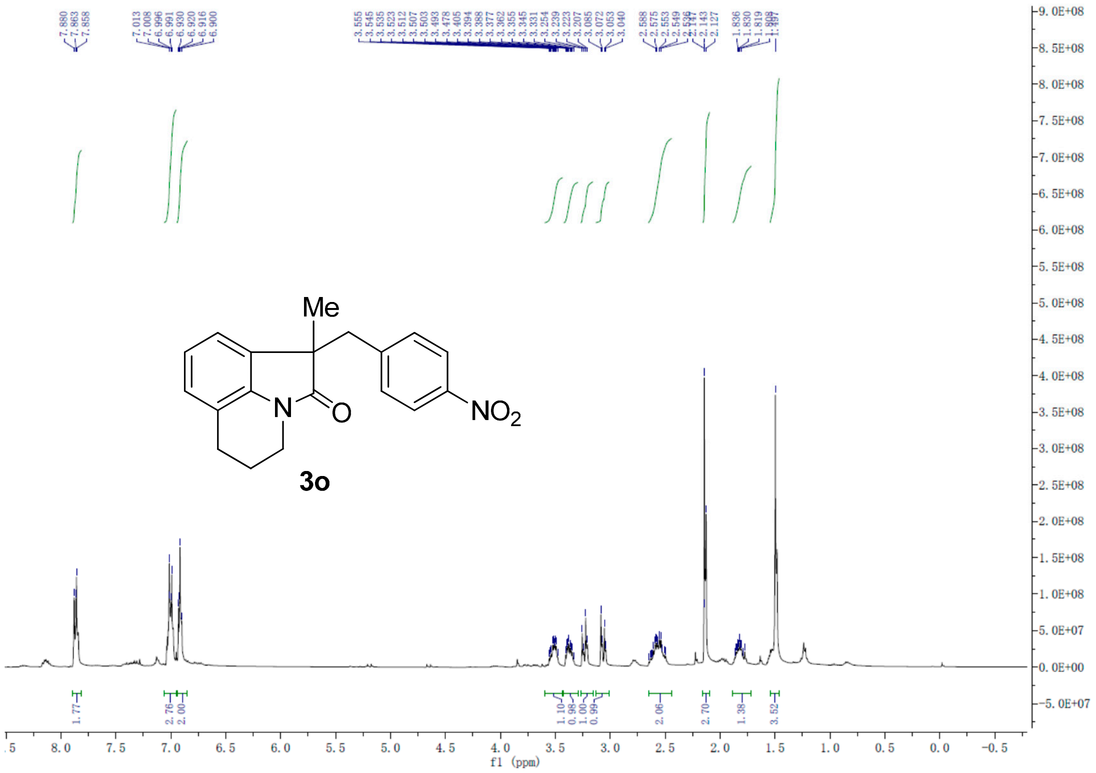

Figure S29. ^{1}H-NMR spectra of compound **3o**.

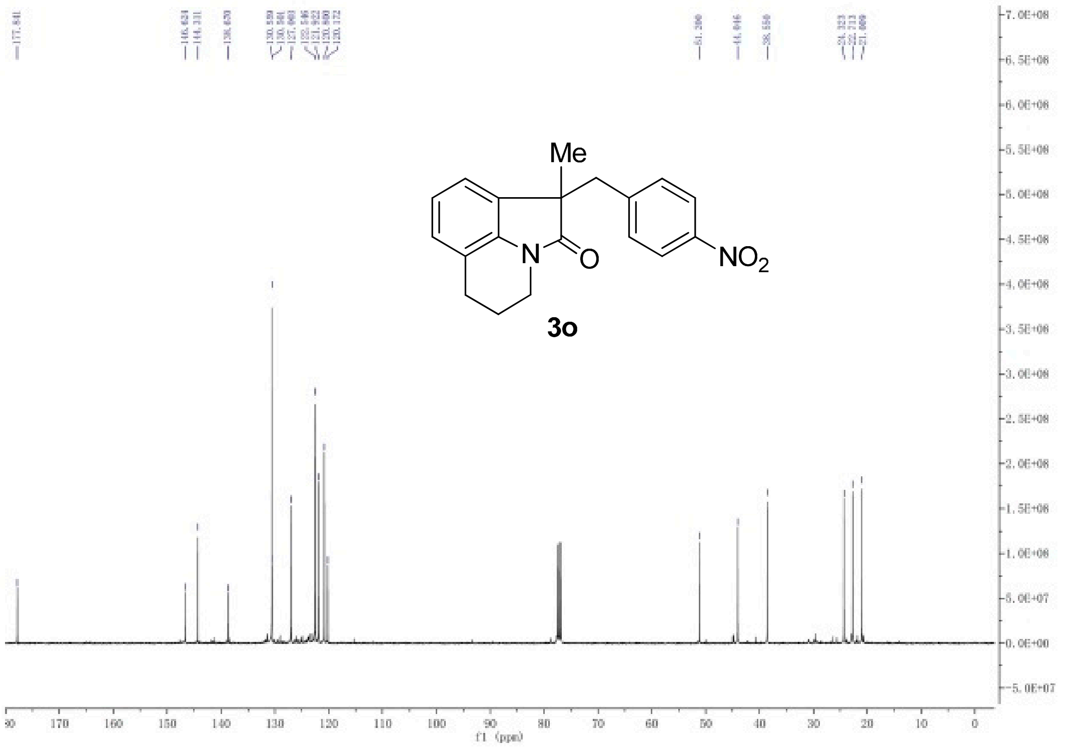

Figure S30. ^{13}C-NMR spectra of compound **3o**.

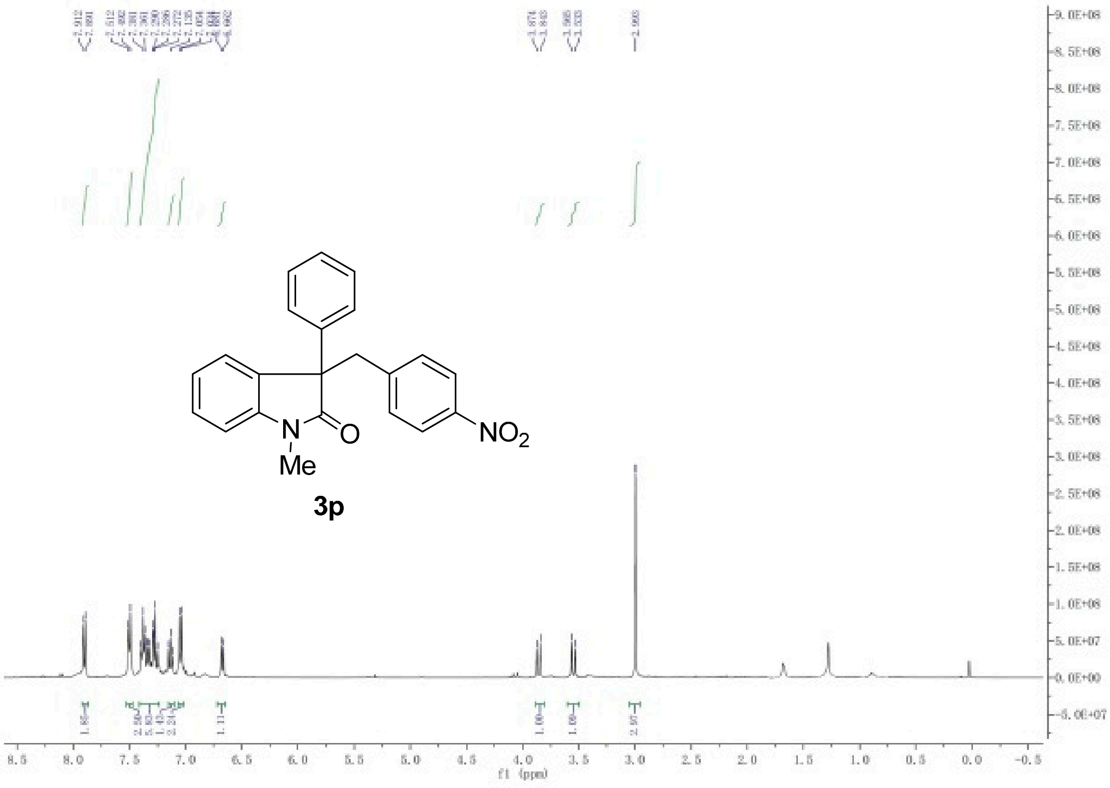

Figure S31. ^{1}H-NMR spectra of compound **3p**.

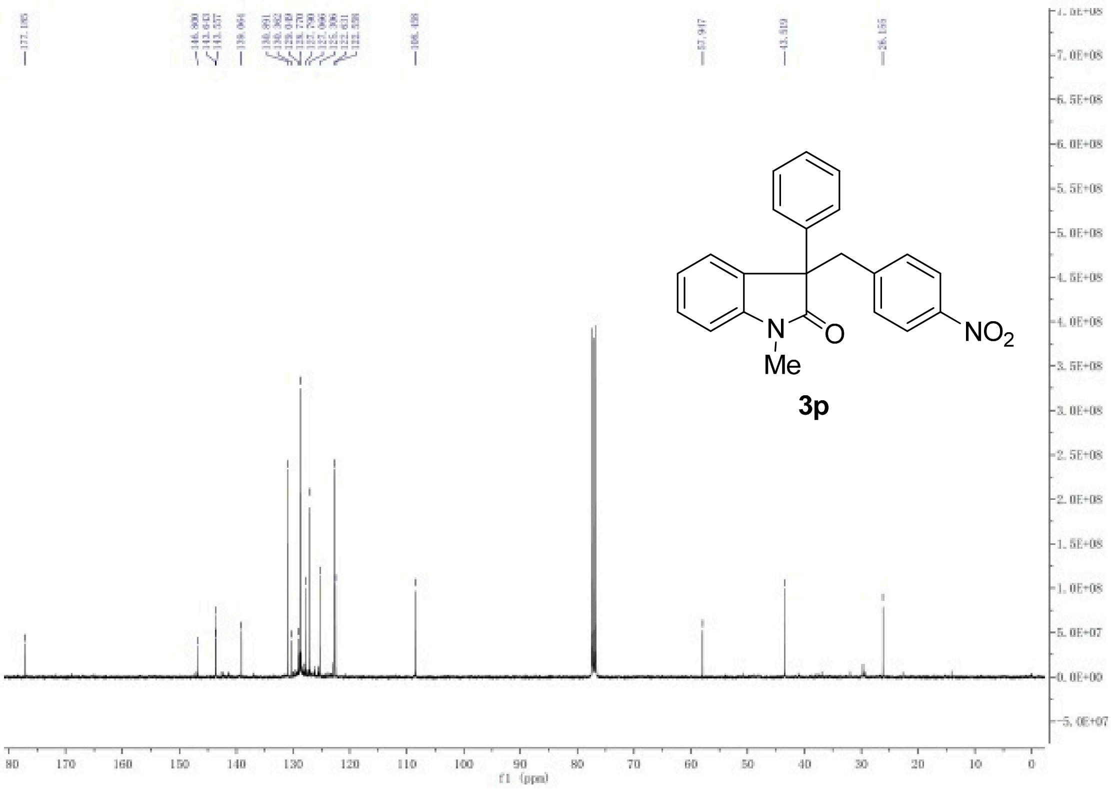

Figure S32. ^{13}C-NMR spectra of compound **3p**.

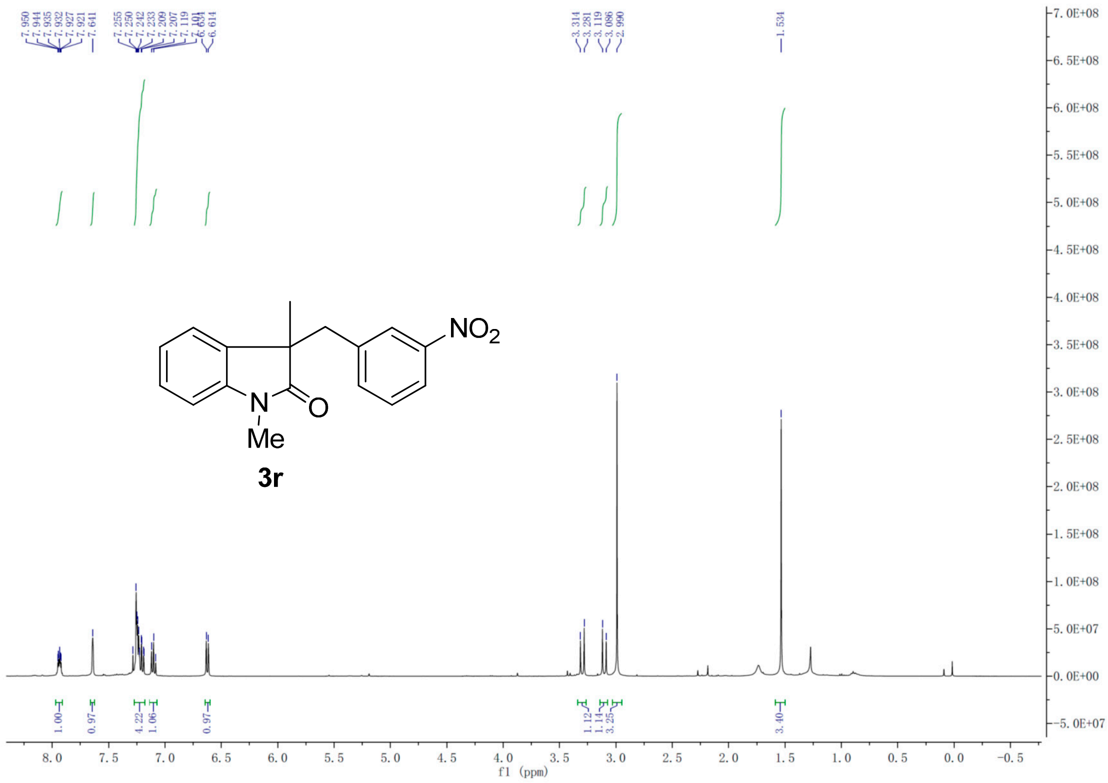

Figure S33. ^{1}H-NMR spectra of compound **3r**.

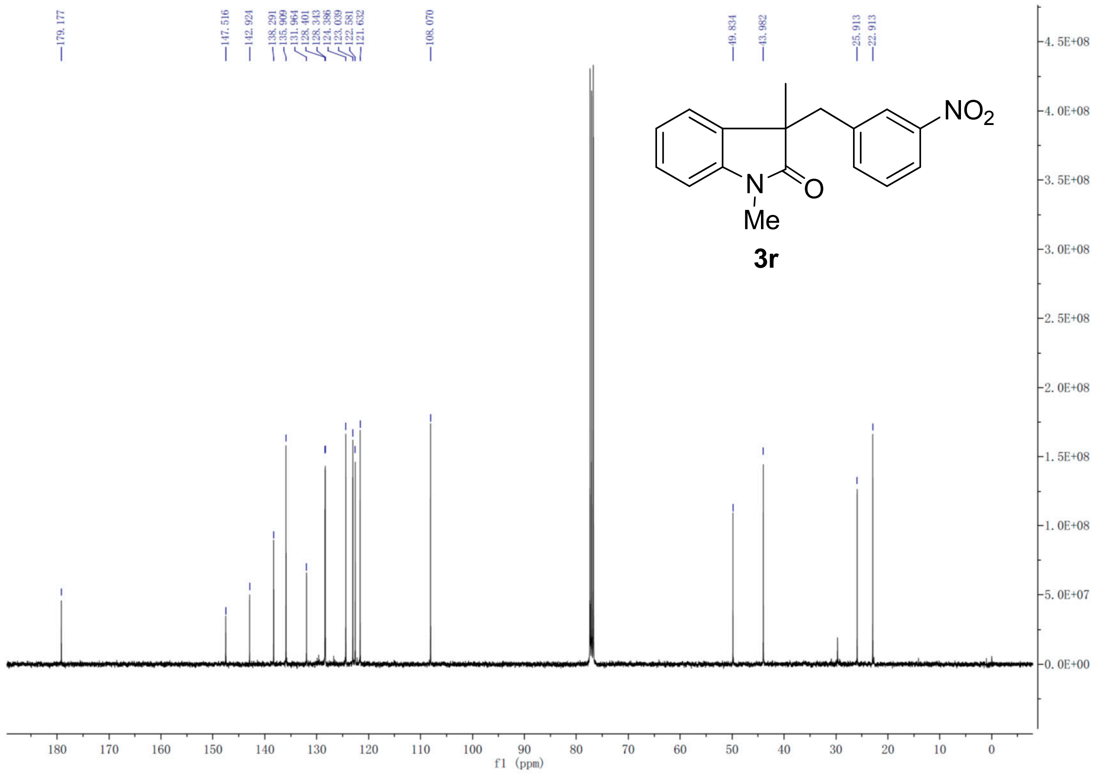

Figure S34. ^{13}C-NMR spectra of compound **3r**.
